# Catalytic Deoxygenation of Hydrolyzed Oil of Chlorella Vulgaris Microalgae over Lanthanum-Embedded HZSM-5 Zeolite Catalyst to Produce Bio-Fuels

**DOI:** 10.3390/molecules27196527

**Published:** 2022-10-02

**Authors:** Mustafa Jawad Nuhma, Hajar Alias, Muhammad Tahir, Ali A. Jazie

**Affiliations:** 1 Department of Chemical Engineering, School of Chemical and Energy Engineering, Universiti Teknologi Malaysia, Johor Bahru 81310, Malaysia; 2 Chemical Engineering Department, College of Engineering, University of Al-Qadisiyah, Al-Diwaniyah City 999048, Iraq; 3 Chemical and Petroleum Engineering Department, United Arab Emirates University (UAEU), Al Ain P.O. Box 15551, United Arab Emirates

**Keywords:** Chlorella Vulgaris microalgae, HZSM-5 zeolite, lanthanum, deoxygenation, hydrocarbons, oxygenates

## Abstract

Microalgae is one of the most important sources of green hydrocarbons because it contains a high percentage of lipids and is likely to reduce reliance on fossil fuels. Several zeolite-based catalysts have a short lifetime due to coke-formation deactivation. In this study, a lanthanum-modified HZSM-5 zeolite catalyst for the conversion of crude oil into non-oxygenated compounds (hydrocarbons) and oxygenated compounds has been investigated. The crude oil of Chlorella Vulgaris microalgae was extracted using Soxhlet and converted into hydrolyzed oil (HO) through a transesterification reaction. The experiments were conducted in a batch reactor (300 °C, 1000 rpm, 7 bar of N_2_, the catalyst to the algal HO ratio of 15% (wt.%) and 6 h). The results were organized into three groups: product yield, chemical composition, and carbon number distribution. The liquid products were investigated, including their elemental composition, higher heating value (HHV), atomic ratios of O/C and H/C, and degree of deoxygenation (DOD%). The loading of lanthanum into HZSM-5 zeolite with different loading percentages enhanced the acid sites needed for the algal HO conversion. Among all the synthesized catalysts, 10%La/HZSM-5 produced the highest conversion of the algal HO, the highest yield of hydrocarbons, the highest HHV, and the highest DOD%; those were 100%, 36.88%, 34.16 MJ/kg, and 56.11%, respectively. The enhanced catalytic conversion was due to the presence of lanthanum, which alters the active sites for the desired reactions of catalytic deoxygenation. The main effect of the modification of the parent HZSM-5 zeolite with lanthanum led to adjusting the acidic sites needed to increase the conversion (%) of the algal HO in the catalytic deoxygenation process and thus increase the hydrocarbon yield (%), which in turn led to an increase in the HHV and DOD%. The proposed La-based zeolite composite is promising for different energy applications due to its unique benefits compared to other expensive and less-stable catalysts.

## 1. Introduction

Global research on alternate and sustainable energy sources has been sparked by the limited reserves of fossil fuels and global warming caused by excessive carbon dioxide emissions [1]. The usage of petroleum derivatives derived from fossil crude oil pollutes the environment by emitting greenhouse gases, which leads to serious environmental issues related to global warming [2].

Many researchers have looked into the manufacture of biofuels using various forms of edible and inedible biomass and their cultivation in various methods [3,4,5,6,7,8]. Microalgae can grow in a variety of environments around the world, including Scandinavian soil with low temperatures and desert soil with high temperatures as well as freshwater and saltwater. Microalgae have a high triglyceride content of up to 60%, making them a promising and essential renewable energy source. Due to their high-fat content, Chlorella Vulgaris microalgae has a bright future in this field [9].

Huge investments have been made in the development of liquid biofuels, which may be the sole alternative to traditional transportation fuels in the future [10]. However, since crude bio-oils still include high oxygen atoms, high viscosity, high freezing points, poor heating values, and thermal instability, they cannot be used directly as biofuel [11]. The most prevalent biodiesel is FAME (fatty acid methyl ester), which is manufactured through transesterification. The world’s biodiesel supply grew from 3.900 billion liters in 2005 to 18.10 billion liters in 2010 and exceeded 30 billion liters in 2016. It is expected to reach 41.40 billion liters in 2025 [12].

When used in combination with fossil fuel at levels of more than 20%, engine modifications are required. Furthermore, the high hygroscopicity of the biodiesel-diesel mixture, which is directly related to the presence of oxygenated molecules that facilitate the proliferation of microorganisms during storage and the formation of undesirable solids, is known to cause many problems, including filter clogging. All of these drawbacks, which are mostly due to the high oxygen content of FAME biodiesel, limit its widespread application [13].

Catalytic deoxygenation is considered an alternative method to the hydrodeoxygenation (HDO) process. Decarboxylation, which yields CO_2_, and decarbonylation, which yields CO, both result in a partial loss of the carbon resources contained in the triglyceride feedstock. However, hydrodeoxygenation, which yields H_2_O, can convert most of the carbon resources in the feedstock to hydrocarbons [13]. Additionally, unlike in hydrodeoxygenation, no water is generated, and the catalyst is not deactivated [12]. Several studies have discussed catalytic deoxygenation utilizing different reactants and catalysts with varying operating parameters, such as reaction time, solvent use, and catalyst-to-feed ratio under different inert gases, such as (N_2_, Ar) [14,15,16,17,18].

Zeolite catalysts were said to be the most commonly utilized catalysts in vegetable oil and bio-oil upgrading. Zhang et al. looked at the catalysts utilized in commercial biofuel refining operations [19]. Although the FCC catalyst and HZSM-5 were proclaimed to have the best performance for catalytic cracking of bio-feedstocks, several zeolite-based catalysts have a short lifetime due to coke-formation deactivation. Xu et al. showed that acidic HZSM-5 has also been linked to the creation of coke by dealkylation, cracking, and aromatization processes. Despite the higher gasoline range of hydrocarbons in the liquid, HZSM-5 created more gas products and a lower required liquid percentage [20]. Furthermore, most studies have found that the HZSM-5 catalyst is the most promising zeolite-type catalyst for increasing the hydrocarbon content in catalytic cracking for various bio-oils [21,22,23,24].

The presence of acid sites in the catalyst improves the cracking and abscission of C-C and C-O bonds in the reactants via dehydration, cracking, aromatization, isomerization, oligomerization, decarboxylation, and dealkylation. Acid sites in catalysts also aid in the formation of hydrocarbons and coke [25]. As a result, to optimize hydrocarbon yields while decreasing the probability of additional polymerization of hydrocarbons into coke materials, the catalyst should be designed with appropriate acidity. Coke is generated when heavy molecules are created and deposited on the surface of the catalyst, reducing its catalytic activity [26]. The active acid sites of the HZSM-5 structure are covered, and the pores are blocked during catalytic cracking, resulting in a reduction in activity and selectivity of the hydrocarbons [26]. Low coke depositions may lead to the catalyst lifetime being extended, and it is necessary to overcome the problem of coke production during the catalytic deoxygenation of algal hydrolyzed oil of Chlorella Vulgaris (HO) [21].

Researchers investigated the use of metal modified on HZSM-5 zeolite to fine-tune the design and acidity of the catalyst, resulting in improved hydrocarbon selectivity, decreased coke formation, and enhanced catalyst lifetime. Transition metals, such as nickel (Ni), molybdenum (Mo), zinc (Zn), and iron (Fe), are used as active components in the catalytic upgrading of oxygenates [26,27]. Despite their great performance in upgrading oxygenates to hydrocarbons, transition-metal-modified HZSM-5 catalysts are prone to coke formation [27].

Several researchers studied the catalytic upgrading of pyrolysis vapors using HZSM-5 zeolite frameworks loaded with poor metals, such as Germanium (Ge), Gallium (Ga), Tin (Sn), and Aluminum (Al). They stated that these poor metals had limited thermal stability and produced more coke than unaltered HZSM-5, in addition to being a costly metal [28]. To the best of our knowledge, no study has included the catalytic deoxygenation process for the bio-oils or the FAMEs over lanthanum-modified HZSM-5 zeolite.

The use of rare earth metals to impregnate HZSM-5 improves the catalyst’s thermal stability [29,30]. Furthermore, the exchange of rare earth metal ions in the zeolite framework has aided hydrogen atom transfer, which manipulates hydrocarbon production at the expense of the oxygenated compound yield. To the best of our knowledge, there is no extensive study in the literature on the influence of La/HZSM-5 on algal HO catalytic deoxygenation upgrading. Furthermore, the majority of the literature on altering HZSM-5 with lanthanum has focused on model compound catalytic cracking processes (alcohol [31,32,33], methyl mercaptan [34,35], alkane [36], pyrolysis of bio-mass [37,38], and furans [39]) over La/HZSM-5 catalysts instead of algal HO (FAMEs) as a reactant.

In this work, lanthanum is investigated further as a bi-functional catalyst for the catalytic deoxygenation upgrading of algal HO and is individually loaded with different loading percentages on the parent HZSM-5. Lanthanum-modified HZSM-5 frameworks are further investigated to upgrade oxygenates into hydrocarbons during catalytic deoxygenation and to minimize coke formation. Different lanthanum loadings, such as 5, 10, and 15 (wt.%), are investigated and characterized using different techniques, such as X-ray diffraction (XRD), nitrogen-adsorption isotherms, scanning electron microscopy (SEM), temperature programmed desorption of ammonia (NH_3_-TPD), and thermogravimetric analysis (TGA).

The catalytic deoxygenation performance for the algal hydrolyzed oil of Chlorella Vulgaris microalgae over all the synthesized catalysts was evaluated in a batch reactor. The influence of lanthanum-loading-weight percentages on the parent HZSM-5 to upgrade the catalytic deoxygenation of the algal HO to convert the oxygenated compounds into hydrocarbons is discussed. The results are organized into three groups: product yield, chemical composition, and carbon number distribution. The liquid product’s characteristics are investigated, including the elemental composition, higher heating value (*HHV*), atomic ratios of O/C and H/C, and degree of deoxygenation (*DOD*%).

## 2. Experimental

### 2.1. Materials

The following materials were used for the synthesis of HZSM-5 and La/HZSM-5 catalysts and used also to extract and hydrolyze the crude oil of Chlorella Vulgaris microalgae as follows: ZSM-5 in Ammonium form [NH_4_^+^] (Si/Al = 30) with a purity of 100% from Alfa Aesar (Haverhill, MA, USA). Lanthanum nitrate hexahydrate (La(NO_3_)_3_·6H_2_O) with a purity of 99.90% is also available from Alfa Aesar (Haverhill, MA, USA). Chlorella Vulgaris microalgae powder was purchased from FocusHerb LLC, Xi’an, China. Methanol with a purity of more than 99.85% from Hayman Company (London, UK), hexane with a purity of 95% from Thomas Baker (Mumbai, India), sulfuric acid with a concentration of 96% from Chem-Lab NV (Zedelgem, Belgium), and sodium hydroxide pellets from Scharlau (Sentmenat, Spain).

### 2.2. Extraction of the Crude Oil from Chlorella Vulgaris Microalgae

The crude oil of Chlorella Vulgaris microalgae was obtained using a Soxhlet extractor system. A Soxhlet extractor apparatus was originally designed for the extraction of a lipid from a solid material. Typically, a Soxhlet extraction is used when the desired compound has limited solubility in a solvent and the impurity is insoluble in that solvent. Chlorella Vulgaris microalgae powder was placed in the algae reservoir, and liquid hexane (200 mL) was added to the hexane/oil reservoir. The hexane fully immerses the microalgae powder and dissolves a small amount of it. When the hexane fills the algae reservoir to a certain level, a siphon is created, and the hexane, along with whatever oil it has dissolved, drains into the hexane/oil reservoir.

Here, a hot plate was set to heat the hexane and the oil mixture at 110 °C. The hexane is boiled to vapor; however, oil has a higher boiling point, and thus it does not vaporize. When the vapor hexane reaches the condenser tube, the cooling water removes heat from the hexane, causing it to condense. The condensed hexane drains to the algae reservoir, where it immerses the microalgae powder and dissolves more oil. The recirculation continues in this way at 110 °C for eight hours. At the end of this extraction process, a mixture of hexane and oil is left in the hexane/oil reservoir and the filtration papers, which contain leftover microalgae powder, are left in the algae reservoir, soaked with residual hexane.

**Figure 1:**
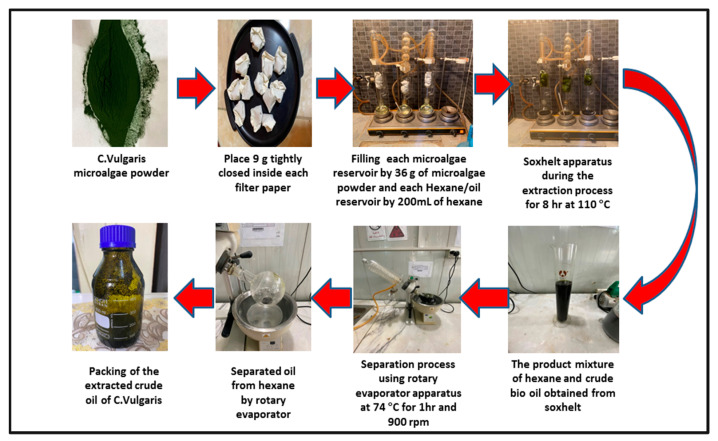
Experimental images for the procedure of extraction of the crude oil from Chlorella Vulgaris microalgae powder.

At the end of the extraction time (8 h), the hexane/oil mixture is collected from the hexane/oil reservoir, kept in the flask, and then sent to the rotary evaporator to separate the hexane from the crude oil of Chlorella Vulgaris microalgae. In the rotary evaporator apparatus, the hexane and oil mixture are placed in the reservoir of the hexane and oil and then heated to 74 °C to remove the hexane to another reservoir (the hexane reservoir), where the vaporized hexane passes through a condenser so that it can be recovered in the hexane reservoir [40]. Figure 1 shows the experimental stages of the extraction process in this study.

### 2.3. Hydrolysis of the Crude Oil of Chlorella Vulgaris Microalgae

**Figure 2:**
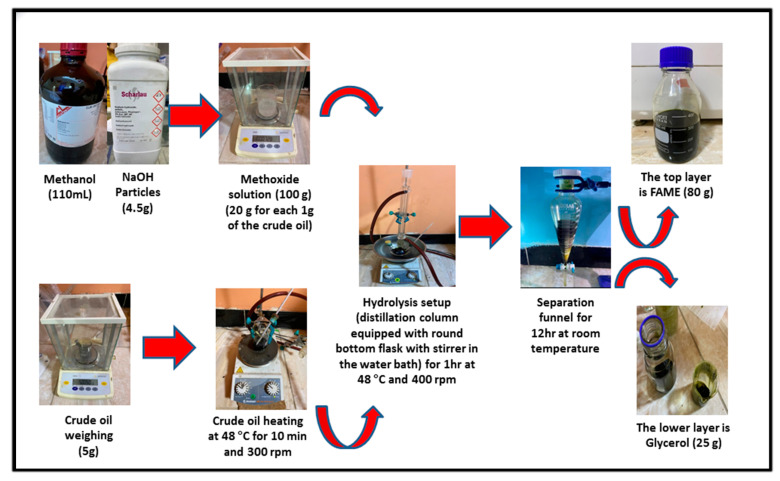
Experimental images for the procedure of hydrolysis of the crude oil of Chlorella Vulgaris microalgae.

The extracted crude oil of Chlorella Vulgaris microalgae was converted to fatty acid methyl esters (FAMEs) (algal hydrolyzed oil of Chlorella Vulgaris microalgae (HO)) using a transesterification reaction. The transesterification reaction was conducted using a hydrolysis setup consisting of a distillation column equipped with a 250 mL round bottom flask with a stirrer. This flask was heated using a water bath as shown in Figure 2. We weighed 5 g of crude oil, and then heated the flask to 48 °C for 10 min while stirring at 300 rpm.

At the same time, to prepare the methoxide (methanol-sodium hydroxide) solution, 110 mL of methanol was added to 4.50 g of solid NaOH and shaken until the complete dissolution of sodium hydroxide particles. Due to the low lipid weight of the crude oil for Chlorella Vulgaris microalgae, the amount of this solution (methoxide) was increased to be around 20 times greater than the crude oil weight. We added 100 g of the methoxide solution to the previously heated crude oil.

This mixture was placed in the round bottom flask and heated to 48 °C for 1 h with stirring at 400 rpm [41]. After the temperature of the flask decreased to room temperature, the mixture was neutralized to PH = 7 using diluted sulfuric acid H_2_SO_4_ (1 M), and then sent to the separating funnel for 10 h. Two layers were formed in the separating funnel. The upper layer was FAMEs, and the lower layer was glycerol. Figure 2 shows the detailed experimental procedure to convert 5 g of the crude oil of Chlorella Vulgaris microalgae to FAMEs.

### 2.4. Catalyst Preparation

To prepare the parent (HZSM-5) zeolite catalyst and lanthanum-modified zeolite catalysts (5% La/HZSM-5, 10% La/HZSM-5, and 15% La/HZSM-5), first, ZSM-5 (NH_4_^+^) in the form of ammonium was converted into a proton (H^+^) form of zeolite (HZSM-5) by the calcination process, where it was calcined at a temperature of 600 °C for 4 h in static air (Ramp rate = 5 °C/min) [28]. For the impregnation of lanthanum rare earth metal on the support (HZSM-5) with different weight percentages, an incipient wetness technique was used along with some modification of the method described by Dueso et al. [42].

To prepare 10 g of 5% La/HZSM-5 catalyst, for example, 9.50 g of HZSM-5 was mixed with 250 mL of deionized water before being mixed with 1.55 g of La(NO_3_)_2_·6H_2_O (lanthanum nitrate hexahydrate) for 1 h at room temperature.

The resultant slurry was heated to 90 °C while stirring at a constant temperature until all the water had evaporated and it became a paste. Next, the paste was dried at 110 °C overnight in an oven and subsequently calcined at 750 °C for 3 h to remove the impurities [43] and then cooled down to room temperature in a desiccator. Finally, a mortar and pestle were used to crush the synthesized catalysts and pack them well to use them later in this study. A similar procedure was used to prepare the other catalysts, 10% and 15% La/HZSM-5, respectively.

### 2.5. Catalyst Characterization

Physicochemical characterizations were conducted to explore the physicochemical characteristics of synthesized catalysts. All the characterization techniques were applied according to standard procedures. The crystallinity of the catalysts was identified using X-ray diffraction (XRD). Powder XRD patterns were obtained using a PW1730 diffractometer (Philips, USA) operated at 40 kV and 30 mA using Cu as an anode material (k = 1.54 Å). The scanning step size was 0.04°/min with 1 s per step in the 5°–100° range (2θ).

Nitrogen-adsorption isotherms were measured at −196 °C using BELSORP-mini II (BEL Japan Inc., Japan). Before analysis, the samples were degassed under vacuum at 200 °C for 6 h to remove adsorbed compounds. The specific surface area and porosity were calculated based on Brunauer–Emmett–Teller (BET) and t-plot models. The total pore volumes were calculated at p/p_0_ = 0.95. The Barrett–Joyner–Halenda (BJH) method was utilized to determine the micropore and mesopore surface area and pore-size distribution.

To characterize the morphology of the zeolite surface and size of the crystals, scanning electron microscopy (MIRA III, TESCAN, Czech Republic) equipped with an energy-dispersive X-ray spectroscopy (EDS) detector (SAMX, Trappes, France) was utilized. The acidity of the samples was determined with the temperature programmed desorption of ammonia (NH_3_-TPD) technique using a NanoSORD-NS91 (Sensiran Co., Tehran, Iran) analyzer.

The possibility of coke forming on the catalysts was measured for all freshly synthesized catalysts using thermogravimetric analysis (TGA) utilizing Q600 (TA, USA) in an air atmosphere at a heating rate of 20 °C/min in the range of 40 °C–800 °C. The thermal gravimetric analysis (TGA) of the parent zeolite HZSM-5 and lanthanum (La)-modified HZSM-5 zeolite catalysts with different loading-weight percentages of La (5, 10, and 15) was performed using a Q600 (TA, USA) instrument in an air atmosphere. Approximately 20 mg of sample was applied to the instrument and held at 40 °C for 1 min. The sample was then heated in dry air at a flow rate of 100 mL/min to 720 °C at a rate of 20 °C/min. The results of the experiments were the temperature dependence of the rate of change in the mass of the sample (dm/dT, %/°C).

### 2.6. Catalytic Deoxygenation of the Algal HO Using the Parent HZSM-5 and Lanthanum-Modified Zeolites

**Figure 3:**
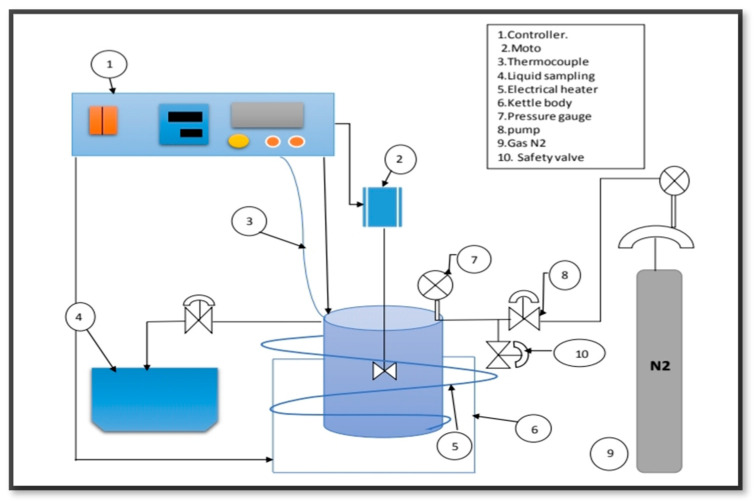
Schematic diagram of the used batch reactor.

In a 100 mL stirring batch reactor (Zhengzhou Keda Machinery and Instrument Co., Zhengzhou, China) (ZZKD), the algae (HO) were catalytically deoxygenated as shown in Figure 3. The algal HO of 23.60 g was mixed with 3.54 g of the catalyst. The mixture was then poured into the reactor. To displace the air, the reactor was closed and squeezed three times with 5 bar of nitrogen (N_2_). The initial N_2_ pressure was then squeezed to 7 bar and retained in the reactor. The reactor was then heated to 300 °C and maintained at that temperature for 6 h, with the impeller rotating at 1000 rpm. The mixes were allowed to cool to room temperature when the reaction was completed. The gaseous phase was released (not studied). Filtration was used to separate the catalyst from the liquid phase.

### 2.7. Product Analysis

The composition of the algal HO and the liquid product was analyzed by gas chromatography-mass spectrometry (GC–MS). The gas chromatography system was an Agilent Technologies 7820A GC System equipped with a mass selective detector GC-5977E MSD in electron ionization (EI) mode at 70 eV. An Ultra Alloy Capillary Column UA-5MS 100% dimethylpolysiloxane column (P/N UA1-30M-1.OF, Frontier Laboratories Ltd., Japan) with an inner diameter of 250 μm, a 0.25 μm film thickness, and a length of 30 m was used. The oven temperature was kept at 45 °C for 1 min before being increased to 300 °C at a rate of 6 °C/min for 40 min.

The National Institute of Standards and Testing (NIST) standard mass spectrum library was utilized to identify the product chemicals from GC–MS. The peak area percentage of the GC–MS chromatogram, which may also be expressed as yield percentage, can be used to compute the relative fraction of product chemicals [44], which was shown to be Equation (1). The conversion percentage for the algal HO was calculated using Equation (2) [45], with the same method described by Katikaneni et al. [46]. The average content (wt%) of *X* (*X* = *C*, *H*, and *O*) was calculated by Equation (3) [47]. The higher heating value (*HHV*) of the product was calculated using Equation (4) [47,48]. Equation (5) is used to compute the degree of deoxygenation (*DOD*%) [49].

(1) $$\mathrm{Yield}\;\left(\%\right)=\frac{\mathrm{Area}\;\mathrm{of}\;\mathrm{the}\;\mathrm{desired}\;\mathrm{product}}{\mathrm{Area}\;\mathrm{of}\;\mathrm{all}\;\mathrm{detected}\;\mathrm{substances}}\times 100$$

(2) $$\mathrm{Conversion}\;\left(\%\right)=\frac{\mathrm{Mass}\;\mathrm{of}\;\mathrm{initial}\;\mathrm{compound}\;\mathrm{in}\;\mathrm{the}\;\mathrm{HO}-\mathrm{Mass}\;\mathrm{of}\;\mathrm{the}\;\mathrm{compound}\;\mathrm{in}\;\mathrm{the}\;\mathrm{product}}{\mathrm{Mass}\;\mathrm{of}\;\mathrm{initial}\;\mathrm{compound}\;\mathrm{in}\;\mathrm{the}\;\mathrm{HO}}\times 100$$

(3) $$\mathrm{wt}.\%\;X=\frac{Mass\;of\;X\;in\;products}{Mass\;of\;products}$$

(4) $$HHV\;\left(\frac{\mathrm{MJ}}{\mathrm{Kg}}\right)=-1.3675+0.3137\;\left(C\right)+0.7009\;\left(H\right)+0.0318\;\left(O\right)$$

(5) $$DOD\%=\frac{Molar\left(\frac{O}{C}\right)of\;the\;algal\;HO\;in\;feed-Molar\;\left(\frac{O}{C}\right)of\;the\;catalytic\;cracking\;products}{Molar\left(\frac{O}{C}\right)of\;the\;algal\;HO\;in\;feed}$$

## 3. Results and Discussions

### 3.1. Catalyst Characterization

#### 3.1.1. XRD Results

**Figure 4:**
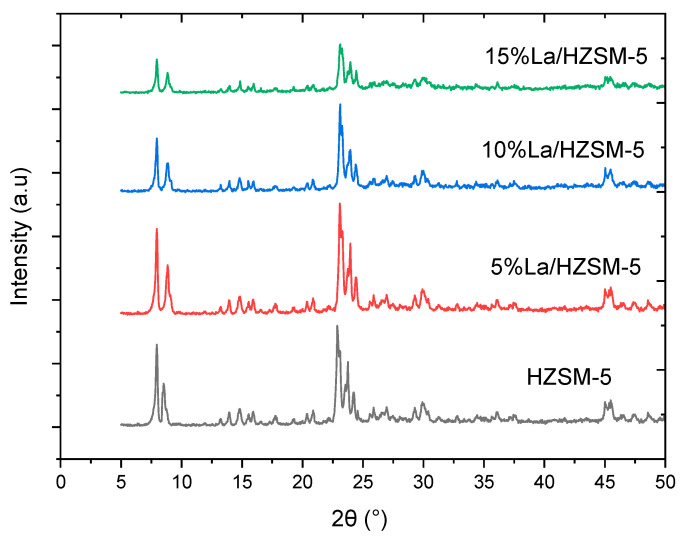
XRD patterns for parent HZSM-5 and lanthanum-modified zeolite catalysts.

X-ray diffraction (XRD) analysis was used to assess the phase purity and crystallinity of the parent HZSM-5, 5% La/HZSM-5, 10% La/HZSM-5, and 15% La/HZSM-5 manufactured catalysts, as shown in Figure 4. The characteristic XRD peaks of HZSM-5 at (2θ = 7.96, 8.52, 14.80, 22.88, 24.24, 29.92, and 45.48°) correspond to the peaks of ZSM-5 of the reference standard for a highly pure calcined ZSM-5. Therefore, the parent HZSM-5 zeolite samples exhibit a typical MFI-type structure (mordenite framework inverted) [32,34,37,50]. The characteristic angles with a small shift from the reference standard peaks were observed. This difference was attributed to the different X-ray sources used [51].

The XRD patterns of 5%La/HZSM-5, 10%La/HZSM-5, and 15%La/HZSM-5 are similar to those of the parent HZSM-5 in the peak positions and shapes, which also indicates that the lanthanum-modified HZSM-5 zeolites have a typical MFI (Mobil fifth) structure [52]. As a consequence, no impurity peaks were found based on the XRD data, and crystalline phases of lanthanum oxides are not detected in the XRD patterns, which indicates that lanthanum oxides may present either as amorphous and highly disperse on the external surfaces of HZSM-5 or penetrate the channels of HZSM-5 as much smaller species [28,52].

As a result, adding 5, 10, and 15% (wt.%) lanthanum using the incipient wetness impregnation method did not affect the parent HZSM-5 framework. This result indicates that all four synthesized zeolites exhibit MFI topology and high purity. However, as compared to the parent HZSM-5 catalyst, the 5% La/HZSM-5 catalyst exhibits higher diffraction peak intensities, and this agrees with the finding of Wei et al. [53].

The differences in the XRD peak intensities for metal-loaded HZSM-5 catalysts could be due to the higher absorption coefficient of the metals during calcination depending upon loaded metal content [54]. The XRD patterns for 10%La/HZSM-5 and 15%La/HZSM-5 show that the peak intensity gradually decreases with increasing lanthanum content, and thus the crystallinity gradually decreases with increasing lanthanum contents.

The phenomenon is possibly due to the increase in the coverage of lanthanum species on HZSM-5 and/or the reduction in the crystallinity with the increase of lanthanum-loading [32]. Generally, the XRD intensities in the pattern of HZSM-5 are sensitive to the presence of any species inside the channels [55]. Therefore, the gradual decrease of peak intensity in the patterns of 10%La/HZSM-5 and 15%La/HZSM-5 implies the entrance of lanthanum species into the channels of the HZSM-5 [39,52,56].

#### 3.1.2. Surface Analysis

Table 1 shows the textural properties, such as the BET surface area, micropore area, external surface area, and pore volume of the parent HZSM-5 and lanthanum-modified HZSM-5 zeolite catalysts with different lanthanum-loading-weight percentage (5, 10, and 15%). From Table 1, we observed that, by increasing the loading-weight percentage of lanthanum on the parent HZSM-5 catalyst, all the values of these textural properties significantly decreased. The BET surface area of the parent HZSM-5 decreased from 338.86 to 293.01, 251.26, and 222.41 m^2^/g for 5%La/HZSM-5, 10%La/HZSM-5, and 15%La/HZSM-5, respectively.

Similarly, the micropore area of the HZSM-5 decreased from 195.24 to 165.96, 162.83, and 157.36 m^2^/g. A similar reduction of the external surface area of the parent HZSM-5 from 143.61 to 127.05, 88.43, and 65.04 m^2^/g. The total pore volume was drastically reduced for the HZSM-5 from 0.22 to 0.19, 0.16, and 0.14 cm^3^/g for 5%La/HZSM-5, 10%La/HZSM-5, and 15%La/HZSM-5, respectively. This decrease in all of these textural properties for the parent HZSM-5 compared to the lanthanum-modified HZSM-5 might be attributed to certain lanthanum oxides accumulating on the pore mouth of the lanthanum-modified zeolite catalysts, as the loading-weight-percent increase as the accumulated lanthanum oxides increase on the pore mouth of the parent catalyst.

Lanthanum oxides, in particular, are smaller in size and may readily diffuse through the pore mouth of HZSM-5 and then deposit within the parent HZSM-5 catalyst’s internal pore channel, thereby, resulting in a considerable drop in the values of all these textural qualities [28,57,58,59]. Moreover, this can also verify the assumption of the decreased XRD intensities that are shown in the XRD pattern [refer to Figure 4] [34].

As shown in Table 1, we observed that the average particle size for the parent HZSM-5 increased with increasing the lanthanum-loading-weight percentage. It was increased for HZSM-5 from 17.70 nm to 20.47, 23.87, and 26.97 nm for 5%La/HZSM-5, 10%La/HZSM-5, and 15%La/HZSM-5, respectively. The surface of the crystal was covered to some extent by the lanthanum loading, and it seems that loaded lanthanum was deposited on the crystal surfaces, which in turn led to an increase in the average particle size of the lanthanum-doped catalysts [36].

**Figure 5:**
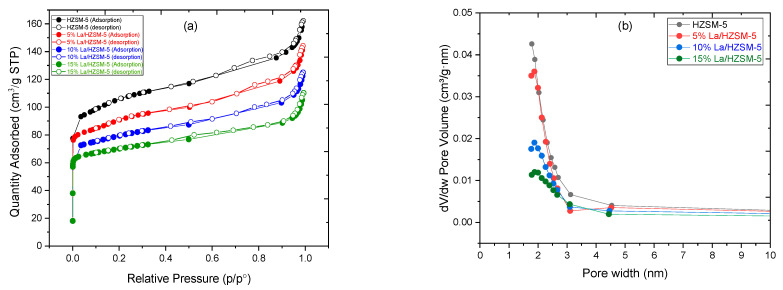
(**a**) N_2_ adsorption–desorption isotherms of the parent HZSM-5 and lanthanum-modified HZSM-5 with different loading-weight percentages and (**b**) pore-size distribution of the parent HZSM-5 and lanthanum-modified HZSM-5 with different loading-weight percentages.

As shown in Figure 5a, based on the IUPAC classification, N_2_ adsorption–desorption isotherms of the lanthanum-modified zeolite catalysts were found to be of type I isotherms with an H_3_ hysteresis loop at a high P/P_0_ region, which is typical of micropore and mesoporous samples [34,60], which is similar to those of the HZSM-5. Moreover, by increasing the lanthanum-loading-weight percentage on the parent HZSM-5 catalyst, the N_2_ adsorption decreased with the relative pressure in the whole pressure range (P/P_0_).

The sharp increase in the adsorption of N_2_ over the samples in the low and medium pressure regions P/P_0_ (0–0.35) confirmed the formation of a microporous zeolite material [33]. The hysteresis loops arise from the change in the uptake of nitrogen (N_2_) at a relative pressure (P/P_0_) in the range of 0.35–0.90, indicating the presence of slit-shaped pores [61,62]. The hysteresis loops arise from the capillary condensation within mesopores via nitrogen multilayers adsorbed on the inner surface [63]. In addition, a small N_2_ uptake step at a relative pressure (P/P_0_) in the range of 0.90–1.00 was observed for both HZSM-5 and lanthanum-modified catalysts, characteristics of interparticle macroporosity [35]. As a result, both micropores and mesopores areas can be found in the parent HZSM-5 and La/HZSM-5-modified catalysts.

Figure 5b shows the pore-size distribution plot obtained from the Harkins and Jura (HJ) model. Figure 5b shows that, for all of the catalyst samples, the pore size was less than 2.50 nm, and revealed by the pore-size distribution, the pore structure was uniform [63]. The high pore size of the parent catalyst enhanced the dispersion of lanthanum on the parent HZSM-5.

#### 3.1.3. Ammonia TPD Analysis

**Figure 6:**
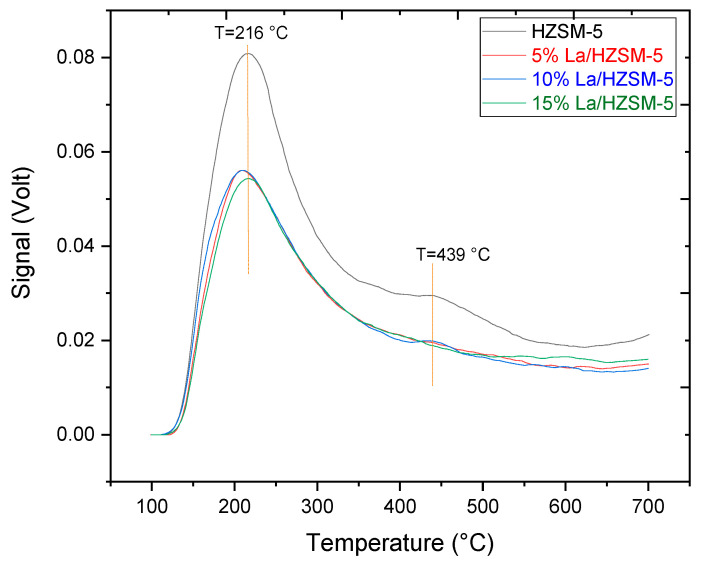
NH_3_-TPD profiles of the parent HZSM-5 and lanthanum-modified catalysts: 5%La/HZSM-5, 10%La/HZSM-5, and 15%La/HZSM-5.

The strong acid sites and weak acid sites on the parent HZSM-5 and lanthanum-modified HZSM-5 zeolite catalysts (5% La/HZSM-5, 10% La/HZSM-5, and 15% La/HZSM-5) have been characterized using NH_3_-TPD [64,65]. Figure 6 exhibits NH_3_-TPD profiles for the parent HZSM-5 and lanthanum-modified HZSM-5 zeolite catalysts. For the parent HZSM-5 catalyst, the typical NH_3_-TPD profile has two maximum peaks at low and high temperatures.

The low-temperature desorption peak at 216 °C was assigned to the desorption of NH_3_ from Lewis sites (weak acid sites) (such as extra-framework aluminum) [28,66,67], while the high-temperature desorption peak at 439 °C was attributed to the desorption of NH_3_ from the Bronsted-acid sites (strong acid sites) deriving from framework aluminum [28,68,69,70].

The peak areas indicated the number of acid sites, and the peak temperature ascribes the acid strength [28] as presented in Figure 6 [35]. In addition, Figure 6 shows that, for all the synthesized lanthanum-modified HZSM-5 zeolite catalysts compared with the parent HZSM-5, when lanthanum loading (wt.%) on the parent HZSM-5 increases, the amount and strength of all the weak and strong acid sites decrease to a different degree, thus, decreasing the total acidity. This is consistent with the results of previous researchers who explained that the modification of zeolites with metals changes the total acidity of the parent zeolite [34,71,72].

The lanthanum species enter into the parent HZSM-5 zeolite tunnel through dispersing or exchanging with H^+^, which would transfer part of the strong Bronsted-acid (B) centers into Lewis-acid (L) centers, thus, altering the surface acidity of the catalysts [73,74,75,76]. Furthermore, acid sites of Lewis type in zeolite catalyst are mostly associated with non-framework (extra-framework) aluminum species [77], while aluminum in the zeolitic framework can cause strong acid sites [78]. Therefore, the increase of the Lewis acid sites’ concentration compared with the Bronsted-acid sites’ concentration in the doped catalysts suggests that the amount of non-framework aluminum species has increased.

The increased amount of non-framework aluminum species can be derived from dealumination in the framework of HZSM-5, as suggested by the XRD results. Accordingly, the dealumination of the framework aluminum species can cause a reduction in the concentration of strong acid sites. Ouyang et al. showed the decrease of the strong acid sites’ concentration would be useful to prohibit the formation of coke deposits. In addition, the decrease in the strong acid sites’ amount was also proved to stabilize the active sites of the catalyst [31].

As illustrated in Figure 6, for all lanthanum-modified zeolite catalysts, the low-temperature peak is slightly shifted to lower temperatures, with a similar peak profile compared with the parent HZSM-5 peak temperatures (at 216 °C). While the high-temperature desorption peak at (439 °C), this peak decreases clearly, and indeed this peak disappears as in the case of 5% La/HZSM-5 and 15% La/HZSM-5.

This is consistent with the results of previous researchers, who explained that the modification of zeolites with metals could lead to the disappearance of the high-temperature desorption peak. They stated that the reason for the disappearance of this peak is due to the presence of metal ions and metal oxides. The number and strength of sites are expected to depend on the content and location (e.g., specific cation exchange sites and extra framework) of the doped metal specie [72,79].

As presented in Table 2, the values of the desorbed NH_3_ (mmol/g) were obtained from the calibration curves of the TCD values in Volt (V). However, the reduction in the total acidities of the lanthanum-modified zeolite catalysts (corresponding to the amount of the weak acid peak with the amount of the strong acid peak) was decreased with increasing the lanthanum-loading-weight percentages compared with the parent HZSM-5, from 0.740 mmol/g for the parent HZSM-5 to 0.512 mmol/g for 15% La/HZSM-5, indicating that loading of lanthanum affects acidic characteristics of the parent HZSM-5 catalysts, which led to the decrease of both strong and weak acid sites amounts.

#### 3.1.4. Thermogravimetric Analysis

**Figure 7:**
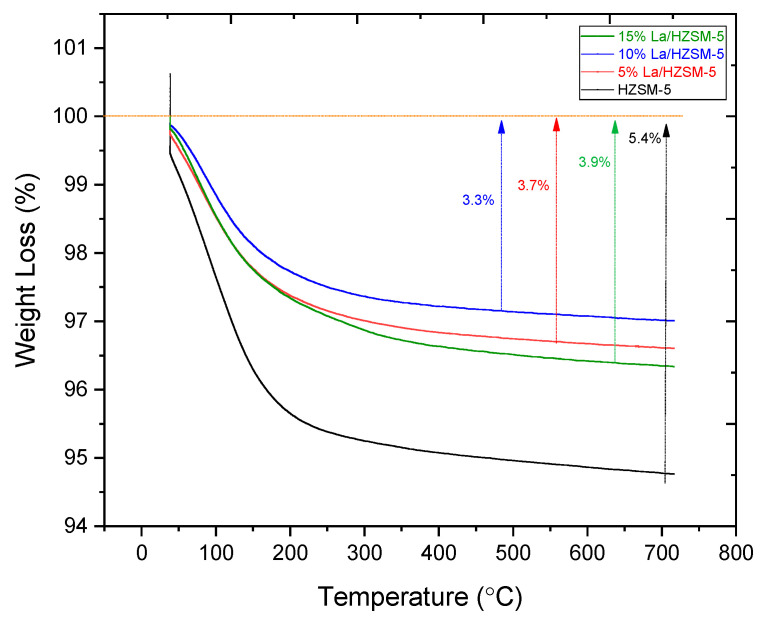
TGA of the fresh parent HZSM-5 and fresh lanthanum-modified HZSM-5 with different loading-weight percentages.

In this study, thermogravimetric analysis (TGA) was utilized to estimate the quantity of carbon that would fill the pores of freshly synthesized catalysts employed in the catalytic deoxygenation process in this study [80]. Figure 7 shows the thermogravimetric analysis (TGA) results for all the fresh synthesized catalysts. It can be observed that the total mass loss for HZSM-5 is 5.40%, while 3.70%, 3.30%, and 3.90% for 5%La/HZSM-5, 10%La/HZSM-5, and 15%La/HZSM-5, respectively, which is apparently due to the release of water from narrow channels [81,82], particularly in the range of (30–170 °C), which was accompanied by water removal from the fresh samples [83]. In conclusion, the addition of lanthanum to the HZSM-5 catalyst decreases the formation ability of coke deposits compared with the parent HZSM-5. The lowest mass loss was on the 10%La/HZSM-5.

#### 3.1.5. SEM Analysis

**Figure 8:**
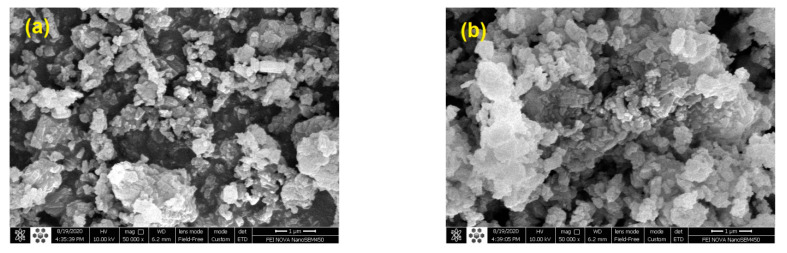

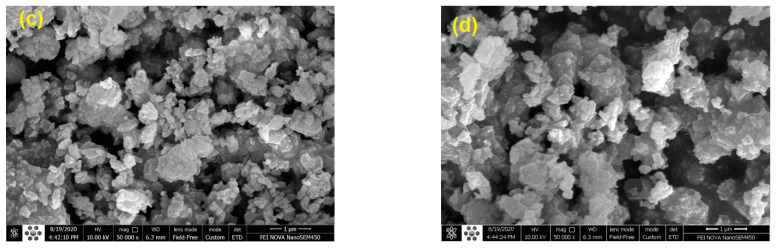
SEM images of HZSM-5 (**a**), 5%La/HZSM-5 (**b**), 10%La/HZSM-5 (**c**), and 15%La/HZSM-5 (**d**).

Figure 8 depicts the surface morphology of the parent HZSM-5 and lanthanum-modified HZSM-5 zeolite catalysts with different lanthanum-loading-weight percentage (5, 10, and 15%). Based on Figure 8, we observed that all the samples exhibit well-crystalline particles of the nanometer scale, which form agglomerates. It is also observed from images that desilication breaks up some of the particles into smaller fragments [84,85].

These fragments can also be seen in the images of lanthanum-modified zeolites, and no significant change in the intrinsic structure is seen after adding rare earth metals [34]. The pure HZSM-5 zeolite catalysts and the lanthanum-modified HZSM-5 zeolite catalysts have identical crystallite shapes and a well-organized structure. Furthermore, the surface of lanthanum-modified zeolite catalysts agglomerated, which might be attributed to the interconnection of tiny particles that happened during the calcination process.

Some small particles are covered on the surface after the modification of lanthanum metal [28,36,86], and these doped samples seem a little rougher than the pure HZSM-5 [28]. However, at this magnification, the distributions of lanthanum particles could not be seen [28,86], which is consistent with the XRD patterns. The average crystallite size of the parent HZSM-5 and lanthanum-modified HZSM-5 zeolite catalysts is about 91–218 nm [28,33,87].

### 3.2. Catalytic Deoxygenation of the HO over the Parent HZSM-5, 5% La/HZSM-5, 10% La/HZSM-5, and 15% La/HZSM-5 Catalysts

To compare the conversion and product composition impacts of all manufactured catalysts used in this investigation, all the experiments were conducted under the same operating conditions in the batch reactor (temperature, time, initial nitrogen pressure, catalyst to HO ratio (wt%.), and stirring), those were (300 °C, 6 h, 7 bar, 15%, and 1000 rpm), respectively. The background of the selection of these operating conditions in this study for catalytic deoxygenation was based on previous studies that related to different types of reactants [26,88,89,90,91].

#### 3.2.1. Conversion of the Algal (HO)

**Figure 9:**
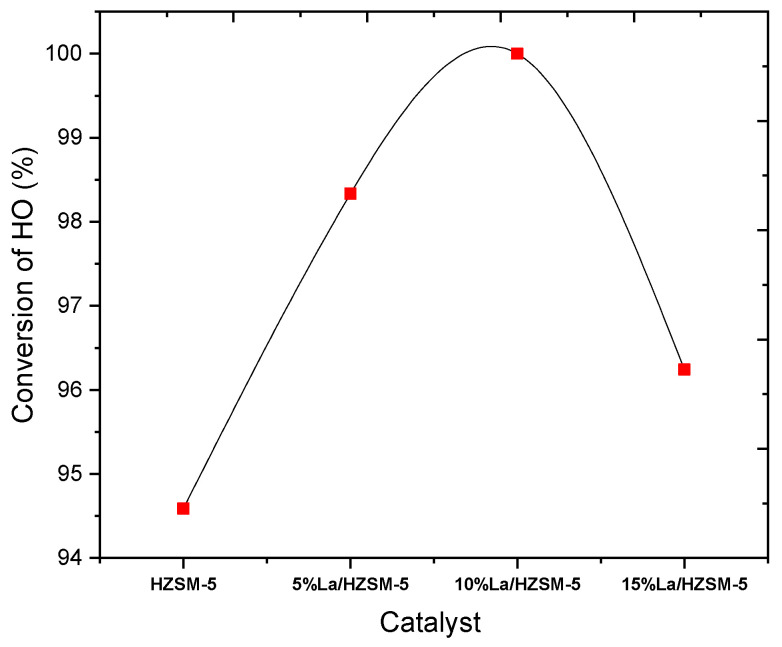
Conversions of the algal (HO) of the catalytic deoxygenation reactions over the parent HZSM-5, 5%La/HZSM-5, 10% La/HZSM-5, and 15%La/HZSM-5 at operating conditions of (batch reactor, 300 °C, 1000 rpm, 7 bar of N_2_, the catalyst to the algal HO ratio = 15% (wt.%) and 6 h).

As shown in Figure 9, and Table 3, the parent HZSM-5 catalyst presented the lowest conversion of the algal HO at 94.589% in all the liquid products of the conducted reactions in this study. This might be attributed to the HZSM-5 catalyst’s efficiency being limited under these reaction conditions. The algal HO conversion percentage was increased in the liquid products using lanthanum-modified zeolite compared to the liquid product using the parent HZSM-5. The conversion percentages of the algal HO were 98.333%, 100%, and 96.242% for the liquid product of the catalytic deoxygenation over 5% La/HZSM-5, 10% La/HZSM-5, and 15% La/HZSM-5, respectively. In conclusion, the loading of lanthanum into HZSM-5 zeolite with different loading percentages enhanced the acid sites needed to improve the algal HO conversion.

The reason for this might be supported by the findings of other studies. To the best of our knowledge, there is no study in literature similar to the catalytic deoxygenation of the algal HO using these catalysts that were used in this study. Therefore, comparisons will be made with close catalytic studies in terms of the operating conditions (such as type of reactor, temperature, time, fatty acids or FAMEs reactants, and the initial pressure of the pumped gas) used in this study.

When Tonya et al. studied the conversion of soybean oil into hydrocarbons by catalytic deoxygenation over Pt/C, Pd/C, and Ni/C under inert gas of nitrogen, they reported that the conversion of soybean oil into hydrocarbons under the same operating conditions depends on the type of catalyst. The conversion percentages were 23%, 30%, and 93% over Pt/C, Pd/C, and Ni/C, respectively [88].

Mathias et al. investigated the stearic acid conversion over twenty different catalysts in the catalytic deoxygenation at 300 °C, 6 bar of Helium. They showed that the conversion percentage of stearic acid depends on the type of catalyst. The highest and lowest conversion percentages were 100% and 4.60% over Pd/C and Ir/SiO_2_, respectively [89].

In addition, Botas et al. showed that the conversion of the rapeseed oil in catalytic cracking of the over HZSM-5, Ni/HZSM-5, and Mo/HZSM-5 in the same operating conditions gives different conversion percentages for the rapeseed oil with different contents of the compounds [26].

In conclusion, the incorporation of lanthanum into the nanocrystalline HZSM-5 zeolite causes important changes in its acid sites and textural properties. The lanthanum oxide is distributed over the zeolite support, being located mainly inside the micropores. The changes induced in the catalyst properties by the metal incorporation also have a strong influence on the catalytic performance.

#### 3.2.2. Chemical Composition Group

**Figure 10:**
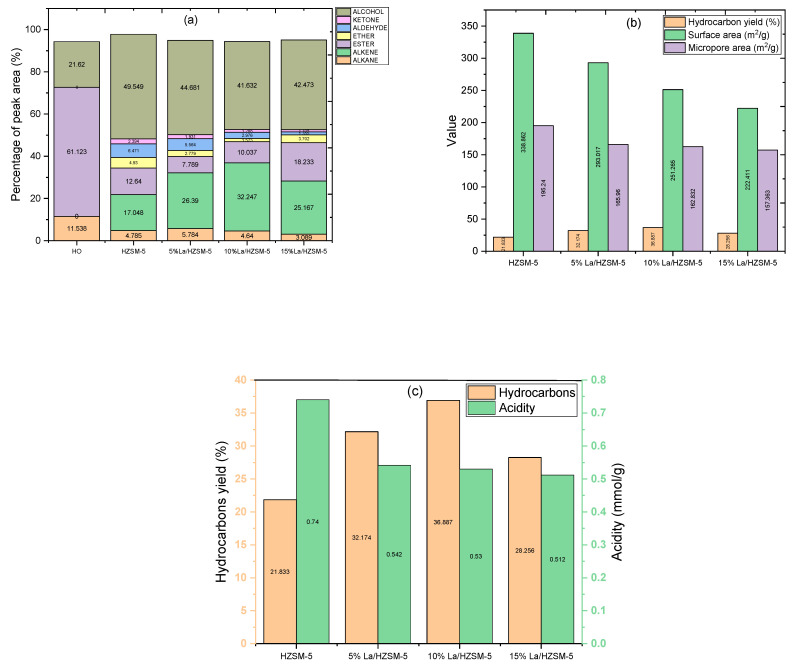
(**a**) The chemical composition groups of the algal HO and the liquid products from the catalytic deoxygenation of the algal HO over the parent HZSM-5 zeolite and lanthanum-modified HZSM-5 zeolite with different loading-weight percentage (batch reactor, 300 °C, 1000 rpm, 7 bar N_2_, catalyst to algal HO ratio = 15% (wt.%) and 6 h), (**b**) hydrocarbon-yield percentage distribution with the surface area and micropore area of the synthesized catalysts, and (**c**) hydrocarbon-yield percentage distribution with the acidity of the synthesized catalysts.

The components and contents of the algal were determined by gas chromatography-mass spectrometry (GC-MS) and quantified by the area normalization method and presented in Figure 10a, and Table 4. Focusing on the algal HO components, they consists of alkane (11.538%), esters (61.123%), and alcohol (phytol) (21.620%). Based on the GC-MS results (refer to Table 4), the yield of the phytol increased in all the conducted experiments in this study, while the yield of alkanes and esters decreased and converted into other compounds (oxygenated compounds and non-oxygenated compounds). In conclusion, the phytol was not a reactant compound; however, the phytol can be considered as a product compound in this study, and the alkanes with the fatty acid methyl esters can be considered as reactant compounds.

The components of the products in this study were divided into seven groups of bio-based chemicals, including two groups of non-oxygenated compounds (alkanes and alkenes) and five groups of oxygenated compounds (esters, ethers, aldehydes, ketones, and alcohols). The composition groups of the algal HO and the liquid products of the catalytic deoxygenation of the HO over all the synthesized catalysts in this study at the operating conditions of 300 °C, 6 h, 15% (catalyst/HO) (weight ratio), initial 7 bar N_2_, and 1000 rpm in a batch reactor are presented in Figure 10a, and Table 4.

Considering the liquid products of catalytic deoxygenation of the algal HO over the parent HZSM-5 zeolite catalyst, the oxygenated compound groups of ether, aldehyde, ketone, and alcohol were increased compared with the other lanthanum-modified zeolite catalysts, which were distributed with 4.93%, 6.47%, 2.39%, and 49.54%, respectively, while the percentage of the ester group was 12.64%. By comparing the products from the parent HZSM-5 catalyst with the other lanthanum-modified zeolite catalysts (5% La/HZSM-5, 10% La/HZSM-5, and 15% La/HZSM-5), the parent HZSM-5 catalyst produced the highest percentage of alcohol (49.54%) and the lowest percentage of the non-oxygenated compounds (21.83%) (hydrocarbons). Those were distributed into 4.78% alkane and 17.04% alkene.

The liquid products from the catalytic deoxygenation of the algal HO over the 5% La/HZSM-5 zeolite catalyst mainly consisted of the alcohol group at 44.68%. This catalyst generated the highest amount of alcohol and the highest amount of aldehyde at 5.56% compared with other lanthanum-modified zeolite catalysts (10% La/HZSM-5 and 15% La/HZSM-5). The other oxygenated compounds of ester, ether, and ketone were produced in percentages of 7.78%, 2.77%, and 1.93%, respectively. The non-oxygenated compounds were produced at 32.17%; those were distributed in 5.78% and 26.39% of alkane and alkene, respectively.

Regarding the second lanthanum-modified zeolite catalyst (10% La/HZSM-5), compared to all synthesized catalysts in this study, the highest amount of hydrocarbons was produced with this catalyst in the percentage of 36.88%. Those were distributed into 4.64% alkane and 32.24% alkene. On the other hand, the lowest amount of ether produced in this study was over this catalyst (10% La/HZSM-5) in the percentage of 1.52%. The amount of aldehyde and ketone were low in the percentages of 2.97% and 1.28%, respectively. Alcohol was produced via this catalyst at a percentage of 41.63%.

In the case of the third lanthanum-modified zeolite catalyst (15%La/HZSM-5), compared to all the synthesized catalysts in this study (HZSM-5, 5% La/HZSM-5, and 10% La/HZSM-5), the highest amount of ester was produced with a percentage of 18.23%, while the hydrocarbons (alkane and alkene) were produced with the lowest percentage compared to the other lanthanum-modified zeolite catalysts (5% La/HZSM-5, and 10% La/HZSM-5) with a percentage of 28.25%. These were distributed in the percentages of 3.08%, and 25.16% for the alkane and alkene, respectively. Alcohol was the dominant oxygenated compound with a percentage of 42.47%. In conclusion, the parent HZSM-5 catalyst produced the highest amounts of oxygenated compounds (ether, aldehyde, ketone, and alcohol), with the lowest amount of hydrocarbons (alkane and alkene) in this study.

In this work, the maximum amount of hydrocarbons was formed using 10% La/HZSM-5. The results also showed that the lowest amounts of ether and alcohol were produced over this catalyst (10% La/HZSM-5). Notably, the highest amount of ester was produced in this study, over 15% La/HZSM-5.

As a result, the catalytic deoxygenation of the algal HO over the parent HZSM-5 zeolite and lanthanum-modified zeolite (5% La/HZSM-5, 10% La/HZSM-5, and 15% La/HZSM-5) catalysts can produce oleochemicals, particularly hydrocarbons and alcohol groups. Those are the important compounds that can be developed to produce biofuels.

Based on Figure 10b, no clear correlation between the physico-chemical properties and yield percentages of the hydrocarbons can be confirmed. The micropore areas and the surface areas for the parent HZSM-5 catalyst were the highest among all the lanthanum-modified zeolite catalysts in this work; however, the yield percentage of the hydrocarbon from this catalyst (HZSM-5) was the lowest in this study (21.833%). For (15%La/HZSM-5), which has the lowest micropore area, and surface area, this catalyst (15%La/HZSM-5) did not produce the highest hydrocarbon yield percentage (28.256%).

Generally, as mentioned previously (refer to Table 1), the micropore areas and the surface areas decreased with increasing the lanthanum loading percentage on the parent HZSM-5 as follows (5%La/HZSM-5 ˃ 10%La/HZSM-5 ˃ 15%La/HZSM-5). The highest yield percentage of hydrocarbon was over 10%La/HZSM-5 (36.887%). In conclusion, it can be concluded that the physical properties of lanthanum-loading percentages on the parent HZSM-5 are not the main reason that triggered the hydrocarbon production. This conclusion is consistent with Zaki et al.; they explained that these physical properties, such as the surface area and micropore area do not affect the effectiveness of the parent catalyst (HZSM-5) for the production of olefins after modifying it with some metals, such as (Cu/HZSM-5 and Ni/HZSM-5) [54].

Figure 10c illustrates the relationship between the hydrocarbon yield percentages with the acidity of all synthesized catalysts in this study. It can be observed that the parent HZSM-5 with the highest total acid sites (0.740 mmol/g) displayed the lowest hydrocarbon yield. However, as mentioned previously, the total acid cites for all the lanthanum-modified zeolite catalysts decreased compared to the parent HZSM-5 [refer to Figure 6 and Table 2]. The total acid sites decreased with increasing the lanthanum-loading percentage on the parent HZSM-5. Those were 0.542, 0.530, and 0.512 (mmol/g) for 5%La/HZSM-5, 10%La/HZSM-5, and 15%La/HZSM-5, respectively.

However, it was noticed that the catalytic deoxygenation performance towards the production of the hydrocarbons varied as a function of the lanthanum-loading percentage on the parent HZSM-5. In general, all of the lanthanum-modified zeolite catalysts yielded higher hydrocarbon yield percentages than the parent HZSM-5 (see Figure 10). In conclusion, the production of hydrocarbons increased as the total acidity decreased compared to the parent HZSM-5 catalyst. On the other hand, by increasing the lanthanum-loading percentage on the parent HZSM-5, the total acidity decreases, and thus the yield percentages of hydrocarbons increases as shown in Figure 10c for 5%La/HZSM-5 and 10%La/HZSM-5.

However, the trend was different for 15%La/HZSM-5, which has the lowest total acid site in this study but did not produce the highest yield of the hydrocarbons. The reason might be that the high lanthanum-loading percentage on the parent HZSM-5 blocked the reactants (particularly the FAMEs) from reaching the active acid sites of the catalyst, thus, decreasing the conversion of the oxygenated compounds of the algal HO into the hydrocarbons. Therefore, this catalyst produced the highest yield percentages of FAMEs in this study (refer to Table 4).

Our results are supported by other studies that discussed the effect of the metal loading on the zeolite catalysts with different reactants and different operating conditions. For example, Li et al. studied the catalytic cracking of the swida wilsoniana oil over different metal loadings of Cu (0, 5, 10, 20, and 30%) on the ZSM-5 (Cu/ZSM-5), and they showed a decrease in the hydrocarbon yield with an increase in the metal loading (Cu) on the parent (ZSM-5) above the value of 10% (such as 20%Cu/ZSM-5, 30%Cu/ZSM-5).

They claimed that the acid sites decreased as the metal loading increased because of the active phase accumulation, which prevented acid site formation [92]. Zhao et al. studied the catalytic cracking of the camelina oil over Zn/ZSM-5 with different loading-weight percentages (10, 20, and 30%), and they showed that the Zn/ZSM-5 with a loading percentage of 30 wt.% Zn blocked fatty acids for decomposition of triglycerides and accelerated catalyst deactivation, which increased the coke yield [93].

Gong et al. studied the coupling conversion of the methanol with the C_4_ alkane over lanthanum-modified HZSM-5 zeolite with different loading metals (0.5, 1.5, 5, and 7%) La/HZSM-5, and they showed that the introduction of lanthanum into HZSM-5 altered the acidity and decreased the strong acid sites (Bronsted-acid sites). They concluded that the influence of the acidity was complicated on the activity of the catalyst towards the production of propylene [32].

#### 3.2.3. The Distribution of Carbon Numbers

For the results from Figure 10a and Table 4, when considering the algal HO conversion into product chemical composition groups, we observed that the catalytic deoxygenation over the synthesized catalysts conditions of initial 7 bar N_2_ pressure, 300 °C, 6 h, 15% weight ratio of catalyst/HO, and 1000 rpm highlighted the high conversion of HO and showed the chemical groups that are intriguing.

To compare the carbon number distributions of the liquid products from these operating conditions with all the synthesized catalysts in this study, the results were presented in terms of carbon number and product content. As mentioned before, there were seven groups of liquid products: two groups for non-oxygenated compounds (alkanes and alkenes) and five groups for oxygenated compounds (esters, ethers, aldehydes, ketones, and alcohols). Non-oxygenated compounds (alkenes) and oxygenated compounds (alcohols) were the main results, as well as small amounts of alkanes, esters, ethers, aldehydes, and ketones were also detected.

The components and contents of the algal HO are shown in Figure 10a and Table 4. The primary chemical groups in algal HO are alkane (11.53%), esters (61.12%), and alcohol (phytol) (21.62%). Based on the GC-MS results (Figure 10 and Table 4), the yield of the phytol increased in all the conducted experiments in this study, while the yield of alkanes and esters were decreased and converted into other compounds (oxygenated compounds and non-oxygenated compounds). In conclusion, the phytol was not a reactant compound; however, the phytol can be considered as a product compound in this study, and the alkanes with the fatty acid methyl esters can be considered as reactant compounds.

**Figure 11:**
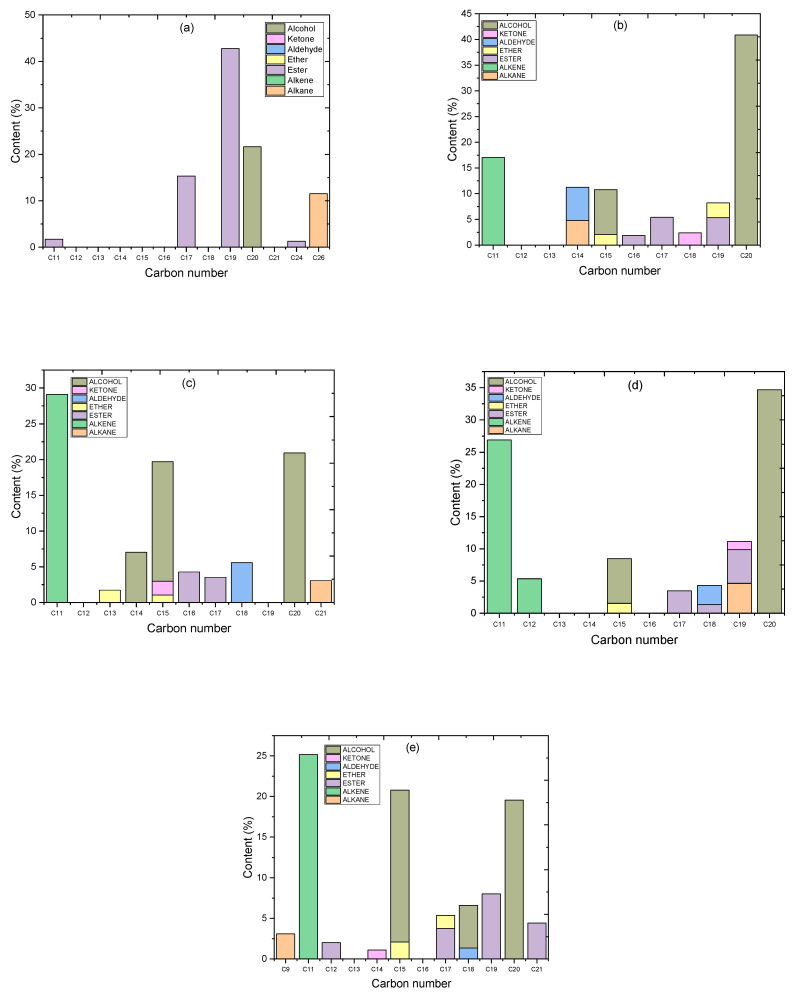
Carbon number distribution for the algal HO (**a**) and the products of the catalytic deoxygenation of the HO over HZSM-5 (**b**), 5% La/HZSM-5 (**c**), 10% La/HZSM-5 (**d**), and 15% La/HZSM-5 (**e**).

As shown in Figure 11a, the esters were the main components of the algal HO (61.12%), were mostly distributed in C_19_ carbon atoms (19) at 42.77%, were distributed 15.08% in 9,12,15-Octadecatrienoic acid, methyl ester, (Z,Z,Z)-(C_19_H_32_O_2_), and 27.69% in 9,12-Octadecadienoic acid, methyl ester (C_19_H_34_O_2_). The other esters were distributed as 15.32% in hexadecanoic acid, methyl ester (C_17_), 1.72% in 6-octen-1-ol, 3,7-dimethyl-, formate (C_11_), and 1.29% in di-n-octyl phthalate (C_24_). The alkane group presented a C_26_ carbon atom (26) at 11.53% in hexacosane (C_26_H_54_). The alcohol chemical group was present in the C_20_ carbon atom at 21.62% in phytol (C_20_H_40_O).

For the liquid product of the catalytic deoxygenation of the algal HO over the parent HZSM-5 zeolite (Figure 11b), the products mainly consisted of the alcohols group (49.54%), which were mostly distributed in C_20_ carbon atoms (20) in the percentage of 40.85% in phytol (C_20_H_40_O), and C_15_ carbon atoms (15) in the percentage of 8.69% in 1-dodecanol, 3,7,11-trimethyl-(C_15_H_32_O). The other main components were non-oxygenated compounds (hydrocarbons) distributed in alkane and alkene.

The total percentage of the hydrocarbons was 21.83% and was mostly distributed in C_11_ carbon atoms at 17.04% in 5-ethyl-1-nonene (C_11_H_22_), and in C_14_ carbon atoms at 4.78% in tetradecane (C_14_H_30_). The other components were with low percentages were for the other chemical groups (esters, ethers, aldehydes, and ketones). The total amount of esters was 12.64% and was distributed into 5.41% C_17_, 1.88% C_16_, and 5.34% C_19_. Those were hexadecanoic acid, methyl ester (C_17_H_34_O_2_); carbonic acid, butyl undec-10-enyl ester (C_16_H_30_O_3_); and trans-13-octadecenoic acid, methyl ester (C_19_H_36_O_2_), respectively.

The ethers group was found in 4.93% of those mostly distributed in 2.84% (C_19_), 1.46% in Disparlure (C_19_H_38_O), and 1.37% in tetrahydropyran 12-tetradecyn-1-ol ether (C_19_H_34_O_2_)], and in 2.08% (C_15_), in oxirane, tridecyl-(C_15_H_30_O). The aldehyde group was 6.47% in (C_14_) in the product tetradecanal (C_14_H_28_O). The ketones group, in the product was found at 2.39% in (C_18_) of 2-pentadecanone, 6,10,14-trimethyl (C_18_H_36_O).

For the liquid product of the catalytic deoxygenation of the algal HO over 5% La/HZSM-5 zeolite [Figure 11c], the products mainly consisted of non-oxygenated compounds (hydrocarbons) with a total percentage of 32.17%, which was mostly distributed in the alkene group with (C_11_) carbon atom of 29.09% that was 5-ethyl-1-nonene (C_11_H_22_); and in the alkane group with (C_21_) that was heneicosane (C_21_H_44_). The other main product for this catalyst (5% La/HZSM-5) is the alcohol group with a percentage of 44.68% mainly distributed in (C_20_) carbon atom of 20.92% Phytol (C_20_H_40_O); (C_15_) in 16.71% of 1-dodecanol, 3,7,11-trimethyl-(C_15_H_32_O); and (C_14_) in 7.03% of Dodeca-1,6-dien-12-ol, 6,10-dimethyl-(C_14_H_26_O).

The esters group was found to be 7.78% in the product was distributed in (C_16_) of 4.27% in carbonic acid, butyl undec-10-enyl ester (C_16_H_30_O_3_); and 3.51% in (C_17_). Those were 1.85%, and 1.66% for pentadecanoic acid, 14-methyl-, methyl ester (C_17_H_34_O_2_); and hexadecanoic acid, methyl ester (C_17_H_34_O_2_), respectively. The ether group was 2.77% was distributed in (C_13_) in 1.73% of 5-Octyn-1-ol tetrahydropyranol ether (C_13_H_22_O_2_) and (C_15_) in 1.04% of Oxirane, tridecyl-(C_15_H_30_O). The aldehyde group was in the liquid product with a percentage of 5.56% in (C_18_) of 13-octadecenal, (Z)-(C_18_H_34_O). The ketone group was 1.93% in C_15_ of 4,7,7-trimethyl-5-(tetrahydropyran-2-yloxy)-bicyclo [2.2.1]heptan-2-one (C_15_H_24_O_3_).

For the liquid product of the catalytic deoxygenation of the algal HO over 10% La/HZSM-5 zeolite [Figure 11d], the products mainly consisted of non-oxygenated compounds (hydrocarbons) with a total percentage of 36.88%, was mostly distributed in the alkene group with (C_11_) carbon atom of 26.90% that was 5-ethyl-1-nonene (C_11_H_22_); and 5.34% in (C_12_) of 1-undecene, 8-methyl-(C_12_H_24_); while the alkane group with 4.64% in (C_19_) that was nonadecane (C_19_H_40_).

The other main product for this catalyst (10% La/HZSM-5) is the alcohol group with a percentage of 41.632% mainly distributed in (C_20_) carbon atom of 34.67% Phytol (C_20_H_40_O); and (C_15_) in 6.95% of 1-dodecanol, 3,7,11-trimethyl-(C_15_H_32_O). The esters group was found at 10.03% in the product and was distributed into 3.48% (C_17_), 1.33% (C_18_), and 5.22% (C_19_). Those were pentadecanoic acid, 14-methyl-, methyl ester (C_17_H_34_O_2_); Valeric acid, tridec-2-ynyl ester (C_18_H_32_O_2_); and 11-octadecenoic acid, methyl ester (C_19_H_36_O_2_), respectively. The ether group was 1.52% in (C_15_) of Oxirane, tridecyl-(C_15_H_30_O). The aldehyde group was in the liquid product with a percentage of 2.97% in (C_18_) of octadecenal (C_18_H_36_O). The ketone group was 1.28% in (C_19_) of Cyclohexanone, 2-[([1,1′-biphenyl]-2-ylamino)methylene]-(C_19_H_19_NO).

For the liquid product of the catalytic deoxygenation of the algal HO over 15% La/HZSM-5 zeolite [Figure 11e], the products mainly consisted of the alcohols group (43.47%), mostly distributed in (C_20_) carbon atoms (20) in the percentage of 22.54% in phytol (C_20_H_40_O); (C_15_) carbon atoms (15) in the percentage of 16.69% in 1-dodecanol, 3,7,11-trimethyl-(C_15_H_32_O); and 4.23% in (C_18_) of 9-Octadecen-1-ol, (Z)-(C_18_H_36_O). The other main components were non-oxygenated compounds (hydrocarbons) with a total percentage of 28.25% were mostly distributed in alkene 25.16% (C_11_), and alkane 3.08% (C_19_). Those were 5-Ethyl-1-nonene (C_11_H_22_); and nonadecane (C_19_H_40_), respectively.

The total amount of esters was 18.23% was distributed into 3.75% (C_17_), 2.02% (C_12_), and 8.01% (C_19_), and 4.43% (C_21_). Those were hexadecanoic acid, methyl ester (C_17_H_34_O_2_); 10-methylundecan-4-olide (C_12_H_22_O_2_); cis-13-octadecenoic acid, methyl ester (C_19_H_36_O_2_); and n-propyl 11-octadecenoate (C_21_H_40_O_2_), respectively. Ethers group was found in 3.70% of those mostly distributed in 2.08% (C_15_) of Oxirane, tridecyl-(C_15_H_30_O); and 1.61% (C_17_) of 2H-Pyran, 2-(7-dodecynyloxy)tetrahydro-(C_17_H_30_O_2_). The total percentage of the aldehyde group was found at 1.355% in (C_14_) of tetradecanal (C_14_H_28_O). The ketones group in the product was found at 1.10% in (C_14_) of 2,4-cyclohexadien-1-one, 3,5-bis(1,1-dimethylethyl)-4-hydroxy (C_14_H_22_O_2_).

#### 3.2.4. Outstanding Bio-Based Chemical Products

The product-yield percentage of the selected outstanding compounds of hydrocarbons (alkanes and alkenes) and alcohol groups from catalytic deoxygenation of the algal HO over the parent HZSM-5 catalyst and lanthanum-modified zeolite catalysts were estimated from Equation (1) and are presented in Table 5.

The higher yield percentages (˂20%) obtained during the catalytic deoxygenation reactions of the algal HO using the parent HZSM-5 and lanthanum-modified zeolite catalysts were non-oxygenated compounds (hydrocarbons) and oxygenated compounds (alcohols) as presented in Table 5. For this reason, these catalysts are interesting for catalytic deoxygenation reactions of FAMEs, even at low nitrogen pressure (7 bar), which implies a lower cost.

**Figure 12:**
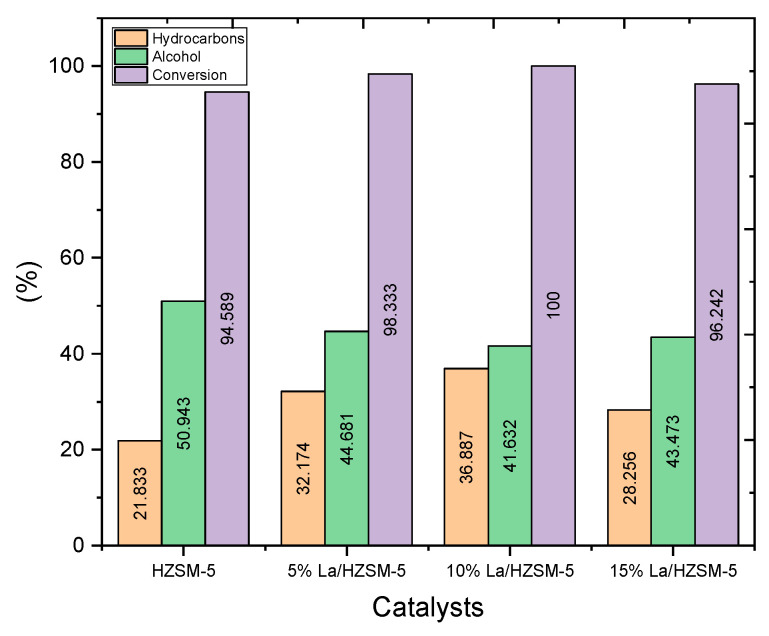
The conversion percentage of the algal HO and the yield percentages of the outstanding chemicals of the hydrocarbons and alcohol from the catalytic deoxygenation of the algal HO over the parent HZSM-5 zeolite and lanthanum-modified HZSM-5 zeolite with different loading-weight percentage (batch reactor, 300 °C, 1000 rpm, 7 bar N_2_, the catalyst to algal HO = 15% (wt.%), and 6 h).

Data from Table 5 and Figure 12 shows that the hydrocarbon yields obtained from the catalytic deoxygenation of the algal HO over all the lanthanum-modified zeolite catalysts were higher than the yields of the hydrocarbons produced by the parent HZSM-5 catalyst. In detail, the total yield of the hydrocarbons of the catalytic deoxygenation of the algal HO over the parent HZSM-5, 5% La/HZSM-5, 10% La/HZSM-5, and 15% La/HZSM-5 were 21.83%, 32.17%, 36.88%, and 28.25%, respectively. The highest yield value for the obtained hydrocarbons was 36.88%, with the highest conversion percentage (100%) of the algal HO in this study was over 10% La/HZSM-5 (refer to Table 3 and Figure 9 for catalytic deoxygenation of the algal HO in the batch reactor at 300 °C, 6 h, 7 bar of initial inert N_2_ gas, and the catalyst to HO ratio = 15% (wt.%).

In addition, the lowest yield of the produced hydrocarbons (21.83%), with the lowest conversion percentage (94.58%) of the algal HO in this study was over the parent HZSM-5. This might be due to prevailing amounts of other oxygenated compounds. For example, the decreased hydrocarbons generated by catalytic deoxygenation of algal HO over the parent HZSM-5 catalyst might be attributable to the predominant generation of alcohols, esters, aldehydes, ketones, and ethers (refer to Table 5).

However, the yield percentages of alkanes in this study are much lower than those of alkenes, and this may be due to the low amount of hydrogen produced during catalytic deoxygenation of the algal HO. It was not sufficient to saturate the double bonds during these reactions. However, this study was conducted under the initial pressure of the inert gas of nitrogen (7 bar) as mentioned previously.

In conclusion, the addition of lanthanum to the parent HZSM-5 zeolite has a significant effect on the total acid sites, particularly on the acidic Bronsted sites. However, this region of strong acid sites (the Bronsted sites) is assumed to be the main catalytic center and acts as dominant acid sites during catalytic deoxygenation reactions [94] as previously explained in the Ammonia TPD discussion.

Table 6 shows the results of different studies related to the catalytic deoxygenation process using different types of catalysts for producing hydrocarbons from different feeds (fatty acids or FAMEs), with operating conditions (reactor type, time, temperature, and the initial value of the charged gas pressure) that are close to the operating conditions in this study. However, our results show lanthanum-modified zeolite presents deoxygenating activity under low pressure of inert gas (nitrogen), which can indicate that the cost of the process would be greatly reduced to produce the hydrocarbons without using hydrogen gas.

Many researchers have discussed catalytic cracking deoxygenation using different catalysts and reactants under initial H_2_ pressure or N_2_ inert gas pressure. Studies related to catalytic deoxygenation under initial inert gas pressure are much less than studies related to the same process under the initial pressure of hydrogen gas. There is no study in literature similar to the catalytic deoxygenation for the algal HO using these catalysts that were used in this study. Therefore, comparisons will be made with close catalytic studies in terms of the operating conditions (such as the type of reactor, temperature, time, fatty acids or FAMEs reactants, and the initial pressure of the pumped gas) used in this study (refer to Table 6).

Focusing on the catalytic deoxygenation under H_2_ gas pressure, Sousa et al. (refer to Table 6) discussed the catalytic deoxygenation of palm kernel oil and the hydrolyzed palm kernel oil over HBeta zeolite under 10 bar of H_2_ as an initial pressure. The yields of the hydrocarbons were 82 ± 3% and 24 ± 9%, respectively. However, they observed that the yields of the hydrocarbons for the catalytic deoxygenation for the olein oil and the hydrolyzed olein oil were 43 ± 3%, and 98 ± 4%, respectively, using the same catalyst with the same operating conditions as shown in Table 6. They claimed that the type of reactants (the volume of reactant molecule, and the length of carbon chain of the reactants) greatly affects the amount of hydrocarbons that are produced with the same catalyst and the same operating conditions [91].

Meller et al. studied the effects of the solvent type and temperature on the catalytic deoxygenation toward the production of the hydrocarbons from the hydrolyzed castor oil using a Pd/C catalyst with an initial 25 bar of H_2_ (refer to Table 6). They explained that the type of solvent used and the temperature of the reaction have a significant impact on the yield percentage of hydrocarbons [95] as presented in Table 6.

Peng et al. studied the catalytic deoxygenation of the stearic acid over 10%Ni/HZSM-5 in the presence of the solvent (dodecane) under 40 bar of initial H_2_ pressure. The total yield of the obtained hydrocarbons was ~56% at 260 °C and 8 h [96] (refer to Table 6). In addition, another study conducted by Peng et al. also studied the catalytic conversion of the microalgae oil over 10%Ni/ZrO_2_ at 270 °C, 40 bar of initial H_2_ pressure in the absence of solvent, and they discussed the effect of reaction time on the total yield of the hydrocarbons. With the same operating conditions, the total yield of the hydrocarbons at 6 h and 4 h were 72% and 61%, respectively [97], (as presented in Table 6).

Duongbia et al. explained the effect of solvent on the catalytic deoxygenation of the palmitic acid over Ni/LY char catalyst under 30 bar of initial H_2_ pressure at 300 °C and 5 h. The highest yield of the hydrocarbons was 12.750% using hexane as a solvent [98], (refer to Table 6).

**Table 6:** Hydrocarbon productions via catalytic deoxygenation with various catalyst types in the references and catalytic deoxygenation in this study.

**Reactant**	**Catalyst**	**Reactant/Catalyst** **Ratio**	**Reactant/Solvent**	**Reactor Type**	**Pressure (bar), Gas**	**Temperature (°C)**	**Time (h)**	**Conversion (%)**	**Observations**	Ref.
palm kernel oil	HBeta zeolite	10/1.50	-	B.R	10 bar H_2_	350	5	-	The total yield of hydrocarbons = 82 ± 3%	[91]
Hydrolyzed palm kernel oil	HBeta zeolite	10/1.50	-	B.R	10 bar H_2_	350	5	-	The total yield of hydrocarbons = 24 ± 9%	[91]
Olein oil	HBeta zeolite	10/1.50	-	B.R	10 bar H_2_	350	5	-	The total yield of hydrocarbons = 43 ± 3%	[91]
Hydrolyzed olein oil	HBeta zeolite	10/1.50	-	B.R	10 bar H_2_	350	5	-	The total yield of hydrocarbons = 98 ± 4%	[91]
Hydrolyzed Macauba oil	HBeta zeolite	10/1	-	B.R	10 bar H_2_	350	5	-	The total yield of hydrocarbons = 30%	[91]
Hydrolyzed castor oil	5% Pd/C	1/0.10	1 g hydrolyzed castor oil/30 mL n-hexane	B.R	25 bar H_2_	310	7	-	The total yield of hydrocarbons = 57%	[95]
Hydrolyzed castor oil	5% Pd/C	1/0.10	1 g hydrolyzed castor oil/30 mL n-dodecane	B.R	25 bar H_2_	310	7	-	The total yield of hydrocarbons = 39.60%	[95]
Hydrolyzed castor oil	5% Pd/C	1/0.10	1 g hydrolyzed castor oil/30 mL n-hexane	B.R	25 bar H_2_	300	7	-	The total yield of hydrocarbons = 40%	[95]
Hydrolyzed castor oil	5% Pd/C	1/0.10	1 g hydrolyzed castor oil/30 mL n-hexane	B.R	25 bar H_2_	340	7	-	The total yield of hydrocarbons ~96%	[95]
Stearic acid	10%Ni/ HZSM-5 (Si/Al = 40)	1/0.20	1 g stearic acid/100 mL dodecane	B.R	40 bar H_2_	260	8		Total selectivity of hydrocarbons ~56%	[96]
Microalgae oil	10%Ni/ HBeta (Si/Al =180)	1/0.20	1 g Microalgae oil/100 mL dodecane	B.R	40 bar H_2_	260	6		The total yield of hydrocarbons = 70%	[96]
Crude oil of Microalgae	10%Ni/ ZrO_2_	1/0.50	-	B.R	40 bar H_2_	270	6	-	The total yield of hydrocarbons = 72%	[97]
Crude oil of Microalgae	10%Ni/ ZrO_2_	1/0.50	-	B.R	40 bar H_2_	270	4	-	The total yield of hydrocarbons = 61%	[97]
Palmitic acid	Ni/LY char	1/1	1 g Palmitic acid/10 g hexane	B.R	30 bar H_2_	300	5	31.410	The total yield of hydrocarbons = 12.75%	[98]
Palmitic acid	Ni/LY char	1/1	1 g Palmitic acid/10 g acetone	B.R	30 bar H_2_	300	5	67	The total yield of hydrocarbons = 12.49%	[98]
Methyl oleate	5%Pd/C	0.83 mol/l/ 1 g of catalyst	-	Semi-batch	15 bar H_2_	300	6	96	Total selectivity of hydrocarbons = 29%	[14]
Methyl oleate	5%Pd/C	0.830 mol/l/ 1 g of catalyst	-	Semi-batch	15 bar Ar	300	6	44	Total selectivity of hydrocarbons = 17%	[14]
Soybean oil	20%Ni /Al_2_O_3_	50/0.550	-	B.R	7 bar N_2_	350	4	74	The total yield of hydrocarbons = 79.50%	[15]
Stearic acid	Pd/ Al_2_O_3_	1	-	B.R	7 bar N_2_	350	6	43	Total selectivity of hydrocarbons = 35%	[16]
Cellulose, and glycerol	HZSM-5 (Si/Al = 36)	cellulose: glycerol: catalyst = 1:0.05:0.004	100 g of n-heptane	B.R	-	350	0.500	-	The total yield of hydrocarbons = 21%	[17]
Cellulose, and glycerol	5%Fe /HZSM-5 (Si/Al = 36)	cellulose: glycerol: catalyst = 1:0.05:0.004	100 g of n-heptane	B.R	-	350	0.500	-	The total yield of hydrocarbons = 38%	[17]
Lauric acid	5%Pd/C	1/0.10	1 g of acid /100 mL of hexadecane	S.B.R	20 bar Ar	300	6	-	The total yield of hydrocarbons = 38	[18]
Lauric acid	5%Pd/C	1/0.10	1 g of acid /100 mL of hexadecane	S.B.R	20 bar Ar	300	3	-	The total yield of hydrocarbons = 28	[18]
Algal HO	HZSM-5 (Si/Al = 30)	1 g of algal HO/0.15 g of the catalyst	-	B.R	7 bar N_2_	300	6	94.58	The total yield of hydrocabons = 21.83%	This study
Algal HO	5%La /HZSM-5 (Si/Al = 30)	1 g of algal HO/0.15 g of the catalyst	-	B.R	7 bar N_2_	300	6	98.33	The total yield of hydrocarbons = 32.17%	This study
Algal HO	10%La /HZSM-5 (Si/Al = 30)	1 g of algal HO/0.15 g of the catalyst	-	B.R	7 bar N_2_	300	6	100	The total yield of hydrocarbons = 36.88%	This study
Algal HO	15%La /HZSM-5 (Si/Al = 30)	1 g of algal HO/0.15 g of the catalyst	-	B.R	7 bar N_2_	300	6	96.24	The total yield of hydrocarbons = 28.25%	This study

Focusing on the catalytic deoxygenation under inert gas pressure. Snare et al. [14], discussed the catalytic deoxygenation of the methyl oleate under the initial reaction atmosphere of H_2_ and Ar. They explained that the type of initial gas pressure has a significant impact on the conversion percentage and the amount of hydrocarbons produced with same of other operating conditions from the catalyst, temperature, feed/catalyst ratio, and time of reaction. The highest conversion percentage and the selectivity of the hydrocarbons were observed under 15 bar of H_2_ compared with 15 bar of Ar (refer to Table 6) [14].

Morgan et al. studied the catalytic deoxygenation of soybean oil over 20%Ni/Al_2_O_3_ under 7 bar of inert gas (N_2_) at 350 °C. The highest hydrocarbon yield percentage was 79.5% with a conversion of 74% [15] (refer to Table 6). In their study, Hollak et al. showed that the total selectivity of hydrocarbons was 35% in the catalytic deoxygenation of stearic acid over Pd/Al_2_O_3_ under 7 bar of nitrogen inert gas pressure at 350 °C and 6 h with a conversion percentage of 43% as presented in Table 6 [16].

Generally, in this study, the catalytic deoxygenation of the algal HO over the parent HZSM-5 produced higher amounts of non-oxygenated compounds in comparison with the lanthanum-modified zeolites. This observation is in line with Li, J. et al. when they used HZSM-5 and 5%Fe/HZSM-5 in the catalyzed liquefaction of cellulose in the presence of the solvent (n-heptane) at 350 °C in a batch reactor. They observed that the parent HZSM-5 gave higher yields of oxygenated compounds and lower yields of non-oxygenated compounds compared with 5%Fe/HZSM-5 [17].

In conclusion, from the above-mentioned studies, the yield percentage of the hydrocarbons that are produced from the catalytic deoxygenation depends on several factors, including temperature, use of solvent, type of solvent, type of reactant, feed/catalyst ratio, reaction time, and the type of initial pressure that is pumped into the reactor before the reaction occurs. Table 6 shows the product-yield percentages of outstanding hydrocarbons (alkanes and alkenes), and alcohols compounds from catalytic deoxygenation of the algal HO over the parent and lanthanum-modified zeolites at 300 °C for 6 h under initial N_2_ pressure of 7 bar, 1000 rpm, and 23.60 g of algal HO/3.54 g of the catalyst in the batch reactor.

Focusing on the alcohol compounds that were produced in this study. The data from Table 5 and Figure 12 show that the alcohol yields produced from the catalytic deoxygenation reactions of the algal HO over all the lanthanum-modified zeolite catalysts were less than the yields of the alcohols produced by the parent HZSM-5 catalyst. In detail, the total yields of the alcohol of the catalytic deoxygenation of the algal HO over the parent HZSM-5, 5% La/HZSM-5, 10% La/HZSM-5, and 15% La/HZSM-5 were 49.54%, 44.681%, 41.632%, and 43.473%, respectively. The highest yield value for the obtained alcohols was 49.54%, with the lowest conversion percentage (94.589%) of the algal HO in this study over the parent HZSM-5 (refer to Table 6 and Figure 9) for catalytic deoxygenation of the algal HO in the batch reactor at 300 °C, 6 h, 7 bar of initial inert N_2_ gas, and the catalyst to the algal HO ratio = 15% (wt.%).

In conclusion, the parent HZSM-5 catalyst produced a higher yield percentage of alcohol, with the lowest yield percentage of hydrocarbons. In addition, 10% La/HZSM-5 produced the lowest yield percentage of alcohol, with a higher yield percentage of the hydrocarbons. This might be due to higher amounts of strong acid sites in HZSM-5 (0.214 mmol/g) than 10% La/HZSM-5 (0.154 mmol/g). Strong acid sites also serve as the primary catalytic center for the catalytic deoxygenation of oxygenated compounds [94]. Thus, a low number of strong acid sites could enhance the upgrading of oxygenates into hydrocarbons.

#### 3.2.5. Liquid Product Characterization

The liquid products were obtained from the catalytic deoxygenation of the algal HO over the parent HZSM-5 and the lanthanum-modified zeolite catalysts at the same operating conditions of 300 °C of reaction temperature, 6 h, 15% (catalyst/HO) (weight ratio), 1000 rpm, and under 7 bar N_2_ in the batch reactor. Equation (3) was used to compute the elemental compositions of liquid products, which are shown in Table 7.

When compared with the algal HO, the products of catalytic deoxygenation from 5% La/HZSM-5, 10% La/HZSM-5, and 15% La/HZSM-5, carbon and hydrogen weight percentages were increasing, whereas the oxygen weight percentages were decreasing. The results of the parent HZSM-5 revealed increasing carbon weight percentages and decreasing hydrogen and oxygen weight percentages. The computed higher heating value (*HHV*) rose when carbon and hydrogen were raised and oxygen was decreased, which is similar to the Jafarian study [99].

As shown in Table 7, compared with the *HHV* (MJ/kg) of HO (32.37), the *HHV* of the catalytic deoxygenation liquid products over the parent HZSM-5, 5% La/HZSM-5, 10% La/HZSM-5, and 15% La/HZSM-5 increased by 33.23, 33.72, 34.16, and 33.67 MJ/kg, respectively. The degree of deoxygenation percentage (*DOD*%) of the liquid products from the modified lanthanum zeolite (10% La/HZSM-5) was greater than the *DOD*s of the liquid products from other synthetic catalysts in this work, as computed from the O/C molar ratios using Equation (3). The elimination of oxygen may enhance the fuel qualities of the products, such as viscosity and acidity [100]. However, when compared to fossil crude oil, the products of all catalysts exhibited low *HHV*s [101].

**Figure 13:**
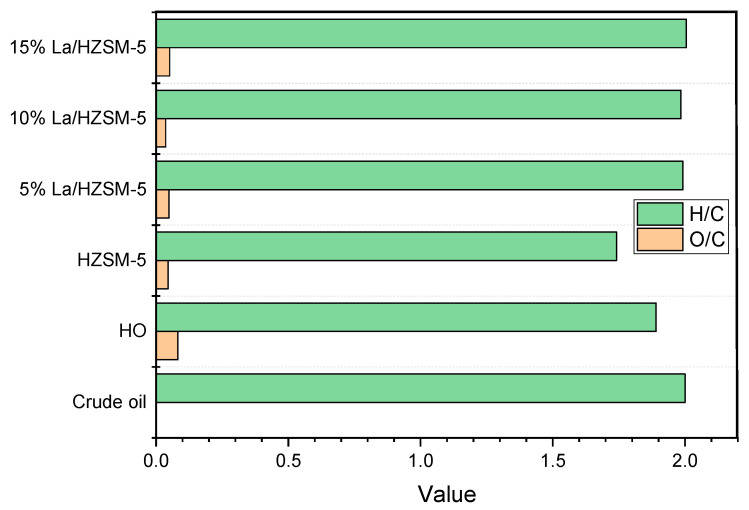
Van Krevelen diagram of the liquid products produced by catalytic deoxygenation of the algal HO over HZSM-5 and lanthanum-modified zeolite catalysts.

Table 7 shows the H/C and O/C atomic ratios, which are displayed as a Van Krevelen diagram (Figure 13). The liquid products of all lanthanum-modified zeolite catalysts exhibited an increasing H/C ratio and a decreasing O/C ratio as compared to the algal HO. In the case of the parent HZSM-5 zeolite, both H/C and O/C atomic ratios were decreased compared with the raw algal HO. For the lanthanum-modified zeolites, the maximum H/C ratio was 2.00 with 15% La/HZSM-5, while the lowest O/C ratio was 0.036 with 10% La/HZSM-5.

Although the H/C ratios of the liquid products were high, the O/C ratios remained high when compared to the O/C ratio of the fossil crude oil (~0). It should be noted that the highest value of *DOD*% was 56.11% over 10% La/HZSM-5 (see Table 7). By comparing this result of this study with the result of a previous study that relates to the hydrotreating of palmitic acid over Ni/LY catalyst under 30 bar H_2_ at 300 °C in a batch reactor, the highest *DOD*% was 65.15%. This is considered low compared with the amount of hydrogen that was used. The highest H/C and *HHV* were 2.03, and 32.40 MJ/kg, respectively, while the highest values of H/C and *HHV* for this study were 2.00 and 34.16 MJ/kg, respectively [98].

## 4. Conclusions

We performed testing of different loading-weight percentages of lanthanum (5%, 10%, and 15%)-impregnated HZSM-5 for catalytic conversion of the algal HO to non-oxygenated compounds and oxygenated compounds by testing the performance in a batch reactor. The lanthanum-loading percentage was responsible for the physical changes that can be observed from the textural properties.

Overall, no direct relationship was deduced from the physical properties. The loading of lanthanum into the HZSM-5 zeolite with different loading percentages changed the acid sites needed for the algal HO conversion. The conversion percentages of the algal HO using all the lanthanum-modified HZSM-5 (in the range of 96.24–100%) were higher than the conversion percentage using the parent HZSM-5 (94.58%). The impregnation of lanthanum into HZSM-5 has an additional effect of generating a higher hydrocarbon yield (28.25–36.88%).

Generally, the increasing performance of catalysts on upgrading algal HO into hydrocarbons was in the following order: 10%La/HZSM-5 > 5%La/HZSM-5 > 15%La/HZSM-5 > HZSM-5. The products of alcohol were in the range of (41.63–44.68%) from the lanthanum-modified zeolite, while from the parent HZSM-5, it was 49.54%. The *DOD*s of the liquid products from lanthanum-modified HZSM-5 catalysts were in the range of 37.60–56.11%, and for liquid products from the parent HZSM-5 catalyst, it was 44.23%.

The *HHV* of the liquid products from all the lanthanum-modified HZSM-5 catalysts (in the range of 33.67–34.16 MJ/kg) was higher than the *HHV* of the liquid product from the parent HZSM-5 (33.23) MJ/kg. Among all the synthesized catalysts in this study, 10%La/HZSM-5 produced the highest conversion of the algal HO, the highest yield of hydrocarbons, the highest *HHV*, and the highest *DOD*%; these were 100%, 36.88%, 34.16 MJ/kg, and 56.11%, respectively.

## Figures and Tables

**Table 1:** The texture properties of the parent HZSM-5 and lanthanum-modified HZSM-5 with different loading-weight percentage-modified HZSM-5.

No.	Catalyst	S_BET_ (m^2^/g)	**S_micro_** **(m^2^/g)**	**S_extern_** **(m^2^/g)**	**V_total_** **(cm^3^/g)**	**V_micro_** **(cm^3^/g)**	Average Particle Size (nm)
**1**	HZSM-5	338.86	195.24	143.61	0.22	0.100	17.70
**2**	5% La/HZSM-5	293.01	165.96	127.05	0.19	0.085	20.47
**3**	10% La/HZSM-5	251.26	162.83	88.43	0.16	0.084	23.87
**4**	15% La/HZSM-5	222.41	157.36	65.04	0.14	0.080	26.97

S_BET_: BET surface area was calculated by Brumauer–Emmett–Teller (BET) mode. S_micro_: Micropore area was determined from the t-plot micropore area. S_extern_: The external surface area was determined from the t-plot area. V_total_: The total pore volumes were obtained from the adsorbed amount at P/P_0_ = 0.95. V_micro_: The micropore volume was measured using the t-plot method.

**Table 2:** NH_3_-TPD properties of HZSM-5, 5%La/HZSM-5, 10%La/HZSM-5, and 15%La/HZSM-5.

Catalyst	Low Peak Temperature Point (°C) (Weak Acid Peak)			High Peak Temperature Point (°C) (Strong Acid Peak)			Total Acid Amount (Total NH_3_ Amount mmol/g)
	T (°C)	TCD (V)	NH_3_ Amount (mmol/g)	T (°C)	TCD (V)	NH_3_ Amount (mmol/g)	
**HZSM-5**	216	0.0808	0.526	439	0.0295	0.214	0.740
**5%** **La/HZSM-5**	207.8	0.0561	0.382	439	0.0195	0.160	0.542
**10%** **La/HZSM-5**	209.5	0.0560	0.376	439	0.0190	0.154	0.530
**15%** **La/HZSM-5**	217.4	0.0543	0.364	439	0.0180	0.148	0.512

**Table 3:** Mass balances (wt.%) of the algal HO compound in the feed, and the compounds of liquid products for the conversion of HO over HZSM-5, 5%La/HZSM-5, 10% La/HZSM-5, and 15%La/HZSM-5.

**Compounds of the Algal** **(HO)**	**Molecular** **Formula**	**Content of the Compound in the Feed (HO)** **(wt.%)**	**Content (wt.%) of the Compound in the Liquid Product of the Catalytic Deoxygenation Reactions for the Algal HO as a Function of the Lanthanum-Loading Percentage on the Parent HZSM-5**			
			**HZSM-5**	**5% La/HZSM-5**	**10% La/HZSM-5**	**15% La/HZSM-5**
Hexacosane	C_26_H_54_	11.53	0	0	0	0
6-Octen-1-ol, 3,7-dimethyl-, formate	C_11_H_20_O_2_	1.72	0	0	0	0
9,12,15-Octadecatrienoic acid, methyl ester, (Z,Z,Z)-	C_19_H_32_O_2_	15.08	0	0	0	0
Hexadecanoic acid, methyl ester	C_17_H_34_O_2_	15.32	5.41	1.66	0	3.75
9,12-Octadecadienoic acid, methyl ester	C_19_H_34_O_2_	27.69	0	0	0	0
Di-n-octyl phthalate	C_24_H_38_O_4_	1.29	0	0	0	0
Phytol	C_20_H_40_O	21.62	40.85	21.92	34.67	22.54
others	-	5.71	0	0	0	0
**Conversion (%) of the HO in the catalytic deoxygenation reactions as a function of the lanthanum-loading percentage on the parent HZSM-5**			**94.58**	**98.33**	**100**	**96.24**

**Table 4:** The main components and content of the algal HO and the products of catalytic deoxygenation reactions for the algal HO over the parent HZSM-5 zeolite and lanthanum-modified HZSM-5 zeolite with different loading-weight percentage (batch reactor, 300 °C, 1000 rpm, 7 bar N_2_, the catalyst to algal HO ratio = 15% (wt.%) and 6 h).

Compound	Molecular Formula	Hydrolyzed Oil (HO)	HZSM-5	5% La/HZSM-5	10% La/HZSM-5	15% La/HZSM-5
**Alkane**						
Hexacosane	C_26_H_54_	11.53				
Tetradecane	C_14_H_30_		4.78			
Heneicosane	C_21_H_44_			3.07		
Nonadecane	C_19_H_40_				4.64	3.08
**Total Alkanes**		**11.53**	**4.78**	**3.07**	**4.64**	**3.08**
**Alkens**						
5-Ethyl-1-nonene	C_11_H_22_		17.04	26.39	21.06	25.16
5-Ethyl-1-nonene	C_11_H_22_			2.70	5.84	
1-Undecene, 8-methyl-	C_12_H_24_				5.34	
**Total Alkens**		**0**	**17.04**	**29.09**	**32.24**	**25.16**
**Esters**						
6-Octen-1-ol, 3,7-dimethyl-, formate	C_11_H_20_O_2_	1.72				
9,12,15-Octadecatrienoic acid, methyl ester, (Z,Z,Z)-	C_19_H_32_O_2_	15.08				
Hexadecanoic acid, methyl ester	C_17_H_34_O_2_	15.32	5.41	1.66		3.75
Carbonic acid, butyl undec-10-enyl ester	C_16_H_30_O_3_		1.88	4.27		
Pentadecanoic acid, 14-methyl-, methyl ester	C_17_H_34_O_2_			1.85		
9,12-Octadecadienoic acid, methyl ester	C_19_H_34_O_2_	27.69				
Di-n-octyl phthalate	C_24_H_38_O_4_	1.29				
trans-13-Octadecenoic acid, methyl ester	C_19_H_36_O_2_		5.34			
Pentadecanoic acid, 14-methyl-, methyl ester	C_17_H_34_O_2_				3.48	
Valeric acid, tridec-2-ynyl ester	C_18_H_32_O_2_				1.33	
11-Octadecenoic acid, methyl ester	C_19_H_36_O_2_				5.22	
10-Methylundecan-4-olide	C_12_H_22_O_2_					2.02
cis-13-Octadecenoic acid, methyl ester	C_19_H_36_O_2_					8.01
n-Propyl 11-octadecenoate	C_21_H_40_O_2_					2.45
n-Propyl 11-octadecenoate	C_21_H_40_O_2_					1.98
**Total Esters**		**61.12**	**12.64**	**7.78**	**10.03**	**18.23**
**Ethers**						
Disparlure	C_19_H_38_O		1.46			
Tetrahydropyran 12-tetradecyn-1-ol ether	C_19_H_34_O_2_		1.37			
Oxirane, tridecyl-	C_15_H_30_O		2.08	1.04	1.52	2.08
5-Octyn-1-ol tetrahydropyranol ether	C_13_H_22_O_2_			1.73		
2H-Pyran, 2-(7-dodecynyloxy)tetrahydro-	C_17_H_30_O_2_					1.61
**Total Ethers**		**0**	**4.93**	**2.77**	**1.52**	**3.70**
**Aldehydes**						
Tetradecanal	C_14_H_28_O		6.47			
13-Octadecenal, (Z)-	C_18_H_34_O			5.56		
Octadecanal	C_18_H_36_O				2.97	1.35
**Total Aldehydes**		**0**	**6.47**	**5.56**	**2.97**	**1.35**
**Ketones**						
2-Pentadecanone, 6,10,14-trimethyl	C_18_H_36_O		2.39			
4,7,7-Trimethyl-5-(tetrahydropyran-2-yloxy)-bicyclo [2.2.1]heptan-2-one	C_15_H_24_O_3_			1.93		
Cyclohexanone, 2-[([1,1′-biphenyl]-2-ylamino)methylene]-	C_19_H_19_NO				1.28	
2,4-Cyclohexadien-1-one, 3,5-bis(1,1-dimethylethyl)-4-hydroxy	C_14_H_22_O_2_					1.10
**Total Ketones**		**0**	**2.39**	**1.93**	**1.28**	**1.10**
**Alcohols**						
1-Dodecanol, 3,7,11-trimethyl-	C_15_H_32_O		4.41	5.32		4.55
1-Dodecanol, 3,7,11-trimethyl-	C_15_H_32_O		4.27	9.18		1.14
1-Dodecanol, 3,7,11-trimethyl-	C_15_H_32_O			1.21		9.66
1-Dodecanol, 3,7,11-trimethyl-	C_15_H_32_O				6.95	1.32
Phytol	C_20_H_40_O	21.62	40.85	21.92	34.67	22.54
Dodeca-1,6-dien-12-ol, 6,10-dimethyl-	C_14_H_26_O			7.03		
9-Octadecen-1-ol, (Z)-	C_18_H_36_O					4.23
**Total Alcohls**		**21.62**	**49.54**	**44.68**	**41.63**	**43.47**
**Total Areas (%)**		**94.28**	**97.79**	**94.91**	**94.34**	**95.12**
**Others Areas (%) = 100-Total Areas (%)**		**5.71**	**2.21**	**5.08**	**5.65**	**4.87**

**Table 5:** The product-yield percentages of outstanding the hydrocarbons (alkanes and alkenes), and alcohols compounds from catalytic deoxygenation of the algal HO over the parent and lanthanum-modified zeolites at 300 °C for 6 h under initial N_2_ pressure of 7 bar, 1000 rpm, and 23.600 g of algal HO/3.540 g of the catalyst in the batch reactor.

Hydrocarbon Compound	Molecular Formula	Hydrolyzed Oil (HO)	HZSM-5	5% La/HZSM-5	10% La/HZSM-5	15% La/HZSM-5
Hexacosane	C_26_H_54_	11.53				
Tetradecane	C_14_H_30_		4.78			
Heneicosane	C_21_H_44_			3.07		
Nonadecane	C_19_H_40_				4.64	3.08
5-Ethyl-1-nonene	C_11_H_22_		17.04	29.09	26.90	25.16
1-Undecene, 8-methyl-	C_12_H_24_				5.34	
**The total yield of the hydrocarbons compounds**		**11.53**	**21.83**	**32.17**	**36.88**	**28.25**
**Alcohol** **Compound**	**Molecular** **Formula**	**Hydrolyzed Oil (HO)**	**HZSM-5**	**5%** **La/HZSM-5**	**10% La/HZSM-5**	**15% La/HZSM-5**
1-Dodecanol, 3,7,11-trimethyl-	C_15_H_32_O		8.69	15.71	6.95	16.69
Phytol	C_20_H_40_O	21.62	40.85	21.92	34.67	22.54
Dodeca-1,6-dien-12-ol, 6,10-dimethyl-	C_14_H_26_O			7.03		
9-Octadecen-1-ol, (Z)-	C_18_H_36_O					4.23
**The total yield of the alcohols compounds**		**21.62**	**49.54**	**44.68**	**41.63**	**43.47**

**Table 7:** The degree of deoxygenation, elemental composition, higher heating values, H/C, and O/C atomic ratios for the algal HO, and the liquid products of the catalytic deoxygenation over the parent HZSM-5 and lanthanum-modified zeolite catalysts.

NO.	Liquid Type	Element (%)			*HHV* (MJ/kg)	H/C (Mole Ratio)	O/C (Mole Ratio)	*DOD*%
		C	H	O				
1	Algal hydrolyzed oil (HO)	78.91	12.43	8.65	32.37	1.89	0.08	n.a
2	Liquid product for HZSM-5	82.90	12.02	5.06	33.23	1.74	0.04	44.23
3	Liquid product for 5% La/HZSM-5	81.20	13.47	5.31	33.72	1.99	0.04	40.25
4	Liquid product for 10% La/HZSM-5	82.40	13.62	3.96	34.16	1.98	0.036	56.11
5	Liquid product for 15% La/HZSM-5	80.93	13.52	5.53	33.67	2.00	0.05	37.60
6	Crude oil [101]	83–86	11–14	˂1	44	1.50–2	~0	n.a

n.a: not applicable.

## Data Availability

Not applicable.

## Sample Availability

Samples of the compounds are not available from the authors.
